# Cardiovascular pharmacotherapy in 2025

**DOI:** 10.1093/ehjcvp/pvag016

**Published:** 2026-04-05

**Authors:** Juan Tamargo, Stefan Agewall, Giuseppe Ambrosio, Claudio Borghi, Elisabetta Cerbai, Gheorghe A Dan, Heinz Drexel, Péter Ferdinandy, Erik Lerkevang Grove, Roland Klingenberg, Joao Morais, William Parker, Bianca Rocca, Patrick Sulzgruber, Anne Grete Semb, Samuel Sossalla, Juan Carlos Kaski, Dobromir Dobrev

**Affiliations:** Department of Pharmacology and Toxicology, School of Medicine, Universidad Complutense, Instituto de Investigación Sanitaria Gregorio Marañón, Avenida Ramón y Cajal s/n, Madrid 29040, Spain; Institute of Clinical Science, Oslo University, Oslo 0318, Norway; Department of Clinical Sciences, Danderyd Hospital, Karolinska Institute, Stockholm 18288, Sweden; Center for Clinical and Translational Research—CERICLET, Department of Medicine, University of Perugia, Perugia 06156, Italy; National Institute for Cardiovascular Research–INRC, Via Irnerio, 48, 40126 Bologna, Italy; Medical and Surgical Sciences Deparment, University of Bologna-IRCCS AOU S. Orsola, Bologna 40138, Italy; Department Neurofarba, Section of Pharmacology and Toxicology, University of Florence, Firenze 50121, Italy; Romanian Academy of Scientists, Carol Davila University of Medicine, Bucharest 050474, Rumania; Vorarlberg Institute for Vascular Investigation & Treatment (VIVIT), Feldkirch 6800, Austria; Department of Pharmacology and Pharmacotherapy, Semmelweis University, Budapest 1089, Hungary; Pharmahungary Group, Szeged 6722, Hungary; Center for Pharmacology and Drug Research & Development, Semmelweis University, Budapest 1085, Hungary; Department of Cardiology, Aarhus University Hospital, Aarhus 8200, Denmark; Department of Clinical Medicine, Faculty of Health, Aarhus University, Aarhus 8200, Denmark; Department of Cardiology, KerckhoffHeart and Thorax Center, Bad Nauheim 61231, Germany; ciTechCare—Center for Innovative Care and Health Technology, Polytechnic University of Leiria, Leira 2414-016, Portugal; Cardiovascular Research Unit, University of Sheffield, Sheffield S5 7AU, UK; Department of Medicine and Surgery, LUM University, Casamassima, Bari 70010, Italy; Department of Medicine, Division of Cardiology, Medical University of Vienna, Vienna 1090, Austria; Preventive Cardio-Rheuma Clinic, Division of Research and Innovation, REMEDY centre, Diakonhjemmet Hospital, Oslo 0370, Norway; Medical Clinic I, Cardiology and Angiology, Gießen & Department of Cardiology, Campus Kerckhoff, Bad Nauheim and Justus-Liebig-Universität, 35392 Gießen, Germany; Molecular and Clinical Sciences Research Institute, St.George’s, University of London, Cranmer Terrace, London SW17 0RE, UK; Institute of Pharmacology, Medical Faculty, University Duisburg-Essen, Essen 45122, Germany; Montréal, Quebec H1T1C8; Department of Medicine, Montreal Heart Institute and Universitéde Montréal, Canada; Department of Integrative Physiology, Baylor College of Medicine, Houston, TX 77030, USA

**Keywords:** Cardiovascular drugs, Cardiovascular pharmacotherapy, Pharmacological strategies

## Abstract

Despite recent advances in cardiovascular pharmacotherapy, prevention and treatment of many cardiovascular diseases remain limited with a clear need for more effective and safer pharmacological strategies. Here, we summarize the most relevant advances in cardiovascular pharmacotherapy in 2025, including the approval of four new drugs (aficamten, etripamil, lerodalcibep, and plozasiran), the label expansions for five already approved drugs, and the results of major randomized clinical trials with already approved drugs, including those that met the prespecified primary endpoints (positive trials) representing new pharmacological options for cardiovascular diseases, those with neutral or negative results, which did not confirm the primary endpoints and the withdrawal from the US market of Andexanet-alfa for safety concerns. Finally, we present the most promising experimental cardiovascular drugs currently being investigated in ongoing Phase 2 and 3 clinical trials.

## Graphical Abstract

**Graphical Abstract:**
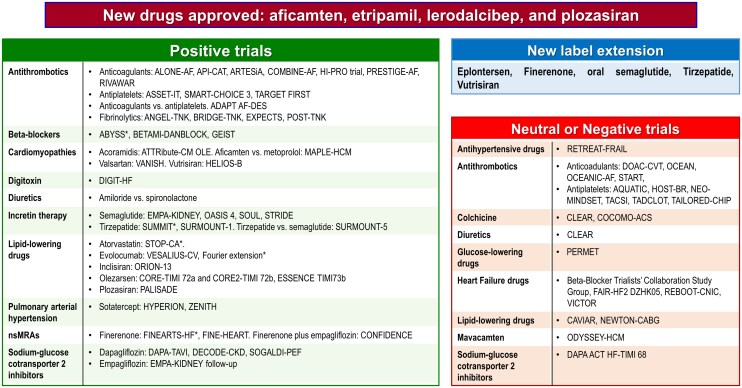
Summary of the most relevant advances in cardiovascular pharmacotherapy in 2025. Abbreviation: SGLT2, sodium-glucose cotransporter 2.

## Introduction

Cardiovascular diseases (CVDs) remain the leading cause of death and disability worldwide. However, despite considerable recent advances in CV pharmacotherapy, the pharmacological treatment of many CVDs is suboptimal and sometimes associated with adverse effects (AEs) and drug–drug interactions. These issues preclude the use of certain drugs in many patients or leads to poor adherence and persistence to treatment, which contribute to worse outcomes. Therefore, there is clear need to develop more effective and safer drugs that prevent or slow the progression of CVDs, improve symptoms and quality of life, and reduce morbidity and mortality.

In 2025, four new CV drugs were approved, four drugs received label extensions, and numerous randomized clinical trials (RCTs) evaluated the efficacy and safety of already marketed drugs and of drugs under clinical development. While some RCTs met their primary endpoints, other RCTs failed to do so. The methodology for selection of the most relevant advances in CV pharmacotherapy in 2025 is summarized in Supplementary material online, *Table S1*.

Here, we review the most relevant clinical trials published in 2025 in the field of CV pharmacotherapy. We first describe the new CV drugs approved and those that obtained an extension of their clinical indications in 2025. Then, we review the clinical trials showing either: (i) superiority of new CV drugs to standard treatment options or to a comparative drug, thus meeting their primary endpoints (i.e. positive trials) (*Table 1*, Supplementary material online, *Tables S2* and *S3*), (ii) no superiority (i.e. negative trials), or (iii) no differences between groups (neutral trials) (see Supplementary material online, *Table S4*). Finally, we summarize Phase 2 and 3 RCTs with CV drugs under clinical development (see Supplementary material online, *Table S1*) and the most important ongoing Phase 2 and 3 clinical trials assessing the efficacy and safety of CV drugs (*Table 2*; Supplementary material online, *Table S5*).

**Table 1:** Clinical trials with positive results^a^

Trial acronym^b^/NCT	Trial/Population	Treatment	Primary endpoint	Results (HR; 95% CI; *P* value)
1. Antithrombotics				
1.1. Anticoagulants				
API-CAT Investigators^1^ NCT03692065	Phase 3, R, DB non-inferiority trial with blinded central outcome adjudication. 1766 with active cancer and proximal DVT or pulmonary embolism who completed at least 6 months of anticoagulant therapy. Female: 56%. FU: 11.8 months	Oral apixaban at a reduced (2.5 mg) or full (5.0 mg) dose bid	Centrally adjudicated fatal or non-fatal recurrent VTE, assessed in a non-inferiority analysis (margin of 2.00 for the upper boundary of the 95% CI of the sub-hazard ratio)	Recurrent VTE, clinically relevant bleeding and mortality occurred in 2.1%, 12.1%, and 17.7% with the reduced-dose, and in 2.8% (*P* = 0.001 for non-inferiority), 15.6% (*P* = 0.03 for superiority), and 19.6% with the full-dose group (*P* = 0.03). The reduced dose decreased the risk of clinically relevant bleeding (0.75; 0.58–0.97; *P* = 0.03). ACD mortality was similar in both groups
PRESTIGE-AF^2^ NCT03996772	Phase 3, OL, R trial. 319 with ICH, AF, indication for anticoagulation, and a score of ≤4 on the mRS. Female: 35%. FU: 1.4 years	DOACs vs. no anticoagulant group (placebo or aspirin, 100 mg od)	First ischaemic stroke and first recurrent ICH. Hierarchical testing for superiority and non-inferiority, respectively, was performed in the intention-to-treat population	DOACs reduced first ischaemic strokes (HR 0.05; 0.01–0.36; *P* < 0·0001) and the rate of all ischaemic stroke events (0.83 vs. 8.60 per 100 patient-years) in survivors of intracerebral haemorrhage with AF. However, for first recurrent intracerebral haemorrhage, the DOAC group did not meet the prespecified HR for the non-inferiority margin of less than 1.73 (*P* = 0.96). DOACs increased the risk of any major bleeding (8.75 vs. 2.04 event rate per 100 patient-years (4.47; 1.82–13.44), ICH (5.00 vs. 2.52 per 100 patient-years) and SAEs compared with the anticoagulation group
1.2. Antiplatelets				
ASSET-IT^3^ NCT06134622	Phase 3, DB, R, PC trial. 832 with acute ischaemic non-cardioembolic stroke who presented within 4.5 h after stroke onset and who were not eligible for thrombectomy. Females: 37%. FU: 90 days	A 24-h IV infusion of tirofiban or placebo within 60 min after IV thrombolysis	An excellent functional outcome (a score of 0 to 1 on the mRS, at 90 days)	At 90 days, a score of 0 to 1 on the mRS was observed in more patients in the tirofiban group than in the placebo group (65.9% vs. 54.9%; RR 1.20; 1.07–1.34; *P* = 0.001). Symptomatic ICH occurred in 1.7% of the patients in the tirofiban group and none in the placebo group, and mortality in 4.1% and 3.8%, respectively.
TARGET FIRST^4^ NCT04753749	OL, P, R, C trial. 1942 with AMI who had undergone complete revascularization with a biodegradable drug-eluting stent within 7 days post-MI and had subsequently completed 1 month of DAPT with no ischaemic or major bleeding events. Females: 21.6%. FU: 11 months	Transition after 1 month to a P2Y12 inhibitor as monotherapy or to continue DAPT for an additional 11 months	Composite of ACD, MI, stent thrombosis, stroke, or major bleeding (BARC type 2–5 bleeding) at 11 months after randomization (tested for non-inferiority with a margin of 1.25% points)	P2Y12-inhibitor monotherapy was non-inferior to continued DAPT with respect to the occurrence of adverse cardiovascular or cerebrovascular events (2.1% vs. 2.2%; *P* = 0.02 for non-inferiority) and resulted in a lower incidence of major bleeding events at 1 year than DAPT (2.6% vs. 5.6%; HR, 0.46; 0.29–0.75; *P* = 0.002 for superiority). Stent thrombosis and the incidence of SAEs was similar in the two groups
2. Fibrinolytics				
BRIDGE-TNK^5^ NCT04733742	Phase 3, OL, 550 with acute ischaemic stroke due to large-vessel occlusion who had presented within 4.5 h after onset and were eligible for thrombolysis. Females: 42%. FU: 90 days	Tenecteplase (IV bolus 0.25 mg/kg; maximum dose, 25 mg) over a period 5–10 s, followed by a saline flush (TNK-thrombectomy) vs. thrombectomy alone	Functional independence (a score of 0 to 2 on the mRS; range, 0 to 6, with higher scores indicating more severe disability) at 90 days	Functional independence was higher in the TNK-thrombectomy group than in the thrombectomy-alone group (52.9% vs. 44.1%; 1.20; 1.01–1.42; *P* = 0.04). There were no differences in symptomatic ICH within 48 h group (6.7% vs. 8.5%; 1.35; 0.74–2.44) and mortality at 90 days (22.3% vs. 19.9%), between the TNK-thrombectomy group and the thrombectomy-alone group respectively
EXPECTS^6^ NCT05429476	Phase 3, P, OL, R trial with blinded outcome assessment. 234 with posterior circulation stroke, without extensive early hypodensity on computed tomography and with no planned thrombectomy. Females: 65%. FU: 90 days	Alteplase (0.9 mg/kg; maximum dose, 90 mg; 10% of the dose as a bolus and the remaining 90% as an infusion over 1 h) or standard medical treatment 4.5 to 24 h after the onset of symptoms	Functional independence (defined as a score of 0 to 2 on the mRS; scores range from 0 to 6, with higher scores indicating greater disability) at 90 days	Alteplase resulted in a higher percentage of patients with functional independence (89.6% vs. 72.6%; aRR 1.16; 1.03–1.30; *P* = 0.01) or no disability (mRS 0–1: 73.9% vs. 60.7%; 1.16; 0.98–1.36). In both groups, the incidence of symptomatic ICH within the first 36 h was similar. At 90 days, 5.2% of patients in the alteplase group and 8.5% in the standard-treatment group had died
3. Beta-blockers				
GEIST Registry^7^ NCT04361994	Open-ended registry. After propensity score matching, two groups of 697 patients with Takotsubo syndrome were paired according to whether they received or not β-blockers at discharge. FU: 2.6 years	Patients paired according to whether or not they received medical therapy with β-blocker at hospital discharge	Impact of β-blocker therapy on long-term mortality and Takotsubo syndrome recurrence	Patients treated with β-blockers had lower mortality rate (6.0 vs. 8.1 per 100 patients/year; 0.71; 0.55–0.90; *P* = 0.006), without differences in Takotsubo syndrome recurrences during follow-up (0.74; 0.61–1.89). There was no association between the use of β-blockers at discharge and LVEF recovery
4. Cardiomyopathies: hypertrophic cardiomyopathy (HCM), transthyretin amyloid cardiomyopathy (ATTR-CM)				
HELIOS-B^8^ NCT04153149	Phase 3, R, PC, DB trial. 655 with either wild-type or hereditary ATTR-CM and a history of HF. The efficacy was assessed in the overall population and in patients not receiving tafamidis (monotherapy population) groups. FU: 42 months	SC vutrisiran 25 mg or placebo every 12 weeks for up to 36 months	Composite of ACD and recurrent cardiovascular events	Vutrisiran reduced the primary end point vs. placebo (overall population: HR 0.72; 0.56–0.93; *P* = 0.01; monotherapy population: 0.67; 0.49–0.93; *P* = 0.02) and ACD (overall population: 0.65; 0.46–0.90; *P* = 0.01). In both populations, vutrisiran compared with placebo improved the 6-MWT (least-squares mean difference, 26.5 m; *P* < 0.001) and KCCQ-OS score (least-squares mean difference, 5.8 points; 2.4–9.2; *P* < 0.001). The incidence of AEs was similar in the two groups
MAPLE-HCM^9^ NCT05767346	Head-to-head, phase 3, DB, DD, R trial. 175 with HCM, resting obstruction (LVOT-G ≥ 30 mm Hg at rest) or latent LVOT-G obstruction ≥50 mm Hg after the Valsalva manoeuvre), symptoms and impaired functional capacity (NYHA class II-III, HF, KCCQ-CSS ≤90), and an age-/sex-predicted pVO_2_ < 100%. Females: 42%. FU: 24 weeks	Aficamten (5 mg up to 20 mg daily) plus placebo or metoprolol (50 mg up to 200 mg daily) plus placebo.	Change from baseline to week 24 in pVO_2_ assessed during cardiopulmonary exercise testing	The change in the pVO_2_ was 1.1 (0.5–1.7) the aficamten group and −1.2 mL (−1.7 to -0.8) mL/kg/min in the metoprolol group (least-squares mean between-group difference, 2.3 mL/kg/min; 1.5–3.1; *P* < 0.001). Patients treated with aficamten had significantly greater improvements in NYHA class, KCCQ-CSS, LVOT-G, NT-proBNP level, and left atrial volume index than patients who received metoprolol (all *P* < 0.01). There were no differences in LV mass index and AEs between treatment groups
5. Digitalis				
DIGIT-HF^10^ EudraCT number, 2013-005326–38	Phase 4, DB, R, PC trial. 1212 with chronic HF (LVEF ≤40% and a NYHA functional class III-IV or a LVEF ≤30% and an NYHA of II). Females: 20%. FU: 3 years	Digitoxin (starting dose of 0.07 mg od, adjusted accordingly the predefined target range of 8–18 ng/mL) to 0.05 or 0.1 mg od or matching placebo	Composite of ACD or hospital admission for worsening HF, whichever occurred first	Digitoxin reduced the primary outcome (0.82; 0.69–0.98; *P* = 0.03) and ACD (0.86; 0.69–1.07; *P* < 0.001 for non-inferiority) vs. placebo but not the total number of deaths from any cause and hospitalizations for worsening HF (0.85; 0.67–1.09; *P* = 0.20). During follow-up, many patients (58.9%) discontinued digitoxin for a substantial period of time
6. Incretin therapy				
SOUL^11^ NCT03914326	Phase 3, DB, PC, event-driven, superiority trial. 9650 with T2D (HbA1C 6.5–10.0%) and ASCVD and/or CKD (eGFR < 60 mL/min/1.73 m^2^). Female: 29%. FU: 47.5 months	Oral semaglutide (3 mg up to 14 mg od) or placebo, in addition to standard care	3-point MACE (composite of CV death, non-fatal MI, or non-fatal stroke), assessed in a time-to-first-event analysis	As compared with placebo, semaglutide reduced the risk of MACE (12% vs. 13.8%; 0.86; 0.77–0.96; *P* = 0.0006) without an increase in the incidence of SAEs and GI disorders. The benefit driven by a reduction in non-fatal MI (26%), non-fatal stroke (12%), and CV death (7%) was consistent across various subgroups.
STRIDE^12^ NCT04560998	Phase 3b, R, DB, PC trial. 792 with T2D and PAD with intermittent claudication (Fontaine stage IIa), an ankle-brachial index ≤0.90 or toe-brachial index ≤0.70. Female: 25%. FU: 52 weeks	SC semaglutide (0.25 up to 1.0 mg) or placebo once per week for 52 weeks	Estimated ratio of the 6-MWD at week 52 when compared with baseline	Semaglutide increased the estimated ratio of median change from baseline in 6-MWD (13%; *P* = 0.0004), pain-free walking distance (*P* = 0.004), and ankle-brachial index (*P* = 0.003), and improved symptoms and QoL (*P* = 0.011) vs. placebo. In a *post hoc* analysis, semaglutide also reduced a composite of rescue therapy, ACD, or major adverse limb adverse events (4% vs. 8%; 0.46; 0.24–0.84)
SUMMIT^13^ NCT04847557	Phase 3, R, DB, PC trial. 731 with NYHA functional class II-IV HFpEF, BMI ≥30 kg/m^2^, and a KCCQ-CSS ≤80 with/without CKD. Female: 54%. FU: up to 104 weeks	SC tirzepatide (2.5 up to 15 mg/week) or placebo	CV death or worsening HF event (assessed in a time-to-first-event analysis) and the change from baseline to 52 weeks in the KCCQ-CSS score	Tirzepatide reduced the composite of CV death or worsening HF vs. placebo (9.9% vs. 15.3%; 0.62; 0.41-0.95; *P* = 0.026), improved health status (KCCQ-CSS: 19.5 vs. 12.7 points; *P* < 0.001) and exercise tolerance (assessed by the 6-MWD: 26.0 m vs. 10.1 m) and decreased BW (−13.9% vs. −2.2%) and high-sensitivity CRP level (−38.8% vs. −5.9%; all *P* < 0.001). AEs (mainly GI) leading to drug discontinuation were more frequent with tirzepatide vs. placebo group (6.3% vs. 1.4%)
SURMOUNT-5^14^ NCT05822830	Phase 3b, OL controlled trial. 751 with obesity (BMI ≥30 or ≥27 and at least one prespecified obesity-related complication) but without T2D. Female: 64.7%. FU: 72 weeks	Maximum tolerated dose of tirzepatide (10 or 15 mg) or of semaglutide (1.7 mg or 2.4 mg) SC once weekly for 72 weeks	Percent change in weight from baseline to week 72	The least-squares mean percent change in BW from baseline to week 72 was −20.2% with tirzepatide and −13.7% with semaglutide, and in waist circumference −18.4 cm with tirzepatide and −13.0 cm with semaglutide (both *P* < 0.001). More participants treated with tirzepatide than with semaglutide had BW reductions of at least 10%, 15%, 20%, and 25% from baseline (*P* < 0.001)
7. Lipid-lowering drugs				
CORE-TIMI 72a and CORE2-TIMI 72b Investigators^15^ NCT05079919 and NCT05552326.	Phase 3, DB, R, PC trials. 1061 with severe hypertriglyceridemia (fasting TG ≥500 mg/dL). FU: 6 months	Olezarsen 50 mg or 80 mg vs. placebo monthly for 12 months	Percent change from baseline in TG levels at 6 months, reported as the difference between each olezarsen dose group and the placebo group	At 6 months, the placebo-adjusted least-squares mean change in TG level was -62.9% and −72.2% with olezarsen in the CORE-TIMI 72a trial and −49.2% and −54.5% in the CORE2-TIMI 72b trial (all *P* < 0.001). Olezarsen reduced TG, apoC3, remnant cholesterol, and non-HDL-C levels and the incidence of acute pancreatitis (85%) vs. placebo (all *P* < 0.001). All changes were sustained at 12 months. Olezarse increased liver-enzyme levels and hepatic fat fraction, and produced thrombocytopenia
ESSENCE TIMI73b^16^ NCT05610280	Phase 3, DB, PC trial. 1349 with either moderate hypertriglyceridaemia (TG levels 150–499 mg/dL) and elevated cardiovascular risk or severe hypertriglyceridaemia (TG level ≥500 mg/dL). Female: 40.3%. FU: 6–12 months	Olezarsen 50 mg or 80 mg vs. placebo monthly	Least-squares mean percentage change in TG level from baseline to 6 months among patients with moderate hypertriglyceridaemia	At 6 months, the placebo-adjusted least-squares mean change in TG level was −58.4% and −60.6% in the olezarsen 50- and 80-mg group, respectively (both *P* < 0.001), and 85.0% and 88.7% of patients on 50- and 80-mg of olezarsen (12.5% in the placebo group) had TG levels <150 mg/dL. SAEs were similar across trial groups.
ORION-13^17^ NCT04659863	Phase 3, DB, PC trial. 12 adolescents (mean age 14.8 years) with HoFH (mean baseline LDL-C 272 mg/dL) on maximally tolerated statin treatment, with or without other lipid-lowering drugs. Female: 69%. FU: 330 days	300 mg of inclisiran sodium or placebo, administered on days 1, 90, and 270	Mean percentage change in LDL-C from baseline to day 330	The placebo-adjusted mean percentage change from baseline to day 330 in PCSK9 was −60.2% and in LDL-C −33.3% (−21.6% in the inclisiran group vs. +11.7% in the placebo group). Changes in apoB, non–high-density lipoprotein cholesterol, and total cholesterol were −23.0%, −32.7%, and −27.8%, respectively. No SAEs, treatment discontinuations for AEs or deaths occurred
PALISADE^18^ NCT05089084	Phase 3, R, PA trial. 75 with persistent chylomicronemia (median levels 2044 mg/dL), with or without a genetic diagnosis. Female: 64%. FU: 12 months	SC plozasiran (25 mg or 50 mg) or placebo every 3 months for 12 months.	Median percent change from baseline in the fasting TG level at 10 months	At 10 months, the median change from baseline in the fasting TG level was −80% and −78% in the 25-mg and 50-mg plozasiran groups and −17% in the placebo group (*P* < 0.001). Plozasiran reduced the incidence of acute pancreatitis (0.17; 0.03− 0.94; *P* = 0.03). The risk of adverse events was similar across groups, but hyperglycaemia with plozasiran occurred in some patients with prediabetes or diabetes at baseline.
VESALIUS-CV^19^ NCT03872401	Phase 3, DB, R, PC trial. 12 257 with atherosclerosis or diabetes and without a previous MI or stroke who had LDL-C levels ≥90 (mean 122) mg/dL. Female: 43%. FU; 4.6 years	SC evolocumab (140 mg) every 2 weeks or placebo	Composite of death from CAD, MI, or ischaemic stroke (3-point MACE) and a composite of 3-point MACE or ischaemia-driven arterial revascularization (4-point MACE)	Compared with placebo, evolocumab significantly reduced 3-point MACE (6.2% vs0.8.0%; 0.75; 0.65–0.86; *P* < 0.001) and 4-point MACE (13.4% vs. 16.2%; 0.81; 0.73–0.89; *P* < 0.001), the composite of CV death, MI, or ischaemic stroke (27%), death from CAD or MI (27%), MI (36%) and ischaemia-driven arterial revascularization (21%) (all *P* < 0.001). No evidence of a between-group difference in the incidence of AEs
8. Pulmonary hypertension drugs				
HYPERION^20^ NCT04811092	Phase 3, DB, PG, R, PC trial. 320 with PAH (WHO functional class II-III), who received the diagnosis <1 year earlier, an intermediate or high risk of death, and treated with double or triple background therapy. Females: 72.5%. FU: 13.2 months	SC sotatercept (starting dose, 0.3 mg/kg uptitrated to 0.7 mg/kg) vs. placebo every 3 weeks	Composite of ACD, unplanned hospitalization (≥ 24 h) for worsening of PAH, atrial septostomy, lung transplantation, or deterioration in performance in exercise testing due to PAH	Sotatercept reduced the risk of clinical worsening (time-to-first-event analysis) vs. placebo (10.6% vs0.36.9%; 0.24; 0.14–0.41; *P* < 0.001) driven by less deterioration in performance in exercise testing due to PAH (5.0% vs. 28.8%) and unplanned hospitalizations for worsening of PAH (1.9% vs. 8.8%), respectively. No cases of atrial septostomy or lung transplantation occurred. Epistaxis and telangiectasia were more frequent with sotatercept than with placebo
ZENITH trial^21^ NCT04896008	Phase 3, DB, R, PC trial. 172 with PAH (WHO functional class III-IV) and high 1-year risk of death (REVEAL Lite 2 risk score, ≥9) who received the maximum tolerated dose of background therapy. Female: 76%. FU: 10.6 months.	SC sotatercept (0.3 mg/kg uptitrated to 0.7 mg/kg) or placebo every 3 weeks	Composite of ACD, lung transplantation, or hospitalization (≥24 h) for worsening PAH, assessed in a time-to-first-event analysis	Sotatercept reduced the risk of a primary end-point event vs. placebo (17.4% vs. 54.7%; 0.24; 0.13- 0.43; *P* < 0.001). Based on this result, the data monitoring committee recommended stopping the trial early for efficacy at an interim analysis. Epistaxis and telangiectasia were the most common AEs in the sotatercept group
9. Non-steroidal mineralocorticoid receptor antagonists				
CONFIDENCE^22^ NCT05254002	Phase 2, DB, R trial. 1664 with chronic kidney disease (eGFR 30 to 90 mL/min/1.73 m2 of body-surface area) with albuminuria (an uACR of 100 to ≤5000 mg/g) and T2D. Females: 25%. FU: 180 days	Finerenone, 10 or 20 mg od (with empagliflozin-matching placebo), empagliflozin 10 mg od (with finerenone-matching placebo), or a combination of finerenone and empagliflozin	Relative change in the log-transformed mean urinary albumin-to-creatinine ratio from baseline to 180 days	The reduction in uCAR with combination therapy was 29% greater than that with finerenone alone (least-squares mean ratio of the difference in the change from baseline 0.71; 0.61–0.82; *P* < 0.001) and 32% greater than that with empagliflozin alone (0.68; 0.59–0.79; *P* < 0.001). AEs and serious AEs leading to discontinuation of the trial treatment occurred in <5% and <2% of the participants in each group.
10. Sodium-glucose cotransporter 2 (SGLT2) inhibitors				
DECODE-CKD^23^ NCT05359263	Phase 2, Single Centre, R, Db, Pc Trial. 222 With CKD (eGFR ≥20 To <60 mL/min/1.73 m^2,^ or ≥ 60 and <90 mL/min/1.73 m^2^ with a UACR ≥200 mg/g or a protein:creatinine ratio ≥300 mg/g) treated with maximally tolerated ACEI or ARB. Female: 29.3%. Fu: 6 months	Dapagliflozin 10 mg OD vs. Placebo	Change in LV mass index	At 6 months, dapagliflozin reduced LV mass index compared with placebo (between-group difference: −8.44 g/m^2^; −11.83 to −5.06; *P* < 0.001); this effect was consistent across various subgroups. There were no differences between the two groups in LVEF, left atrial volume index, LV volumes, NT-proBNP or troponin I at 6 months (all *P* > 0.05)
EMPA-KIDNEY follow-up^24^ NCT03594110	4891 with an eGFR from 20 to <45 mL/min/1.73 m^2^ or an eGFR ≥45 but <90 mL/min/1.73 m2 with an uACR ≥200 mg/g. Female: 34%. FU: 2 years	Empagliflozin (10 mg od) or matching placebo	Composite of kidney disease progression or CV death as assessed from the start of the active-trial period to the end of the post-trial period	During the combined active- and post-trial periods, primary-outcome events occurred less frequently in the empagliflozin than in the placebo group (26.2% vs. 30.3%; 0.79; 0.72–0.87). As compared with placebo, empagliflozin reduced kidney disease progression (0.79; 0.72–0.87), end-stage kidney disease 80.74; 0.64–0.87), and ACD or end-stage kidney disease (0.81; 0.72–0.70). However, empagliflozin had no effect on CV death
SOGALDI-PEF^25^ NCT05676684	Phase 3, P, R, OL, blinded-endpoint cross-over trial. 108 patients with HFmrEF/HFpEF (LVEF >40%), NYHA class II–IV HF symptoms, and elevated NT-proBNP. Each treatment sequence was given for 12 weeks. Female: 57%.	Dapagliflozin and spironolactone vs. dapagliflozin alone	NT-proBNP levels (log-transformed) between groups	Compared with dapagliflozin, combination therapy reduced *Log*NT-proBNP levels −0.11 (−0.22 to −0.01; *P* = 0.035) and more patients reached NT-proBNP reductions ≥20% (OR 2.27; *P* = 0.016). Compared with dapagliflozin, combination therapy reduced SBP (−5.2 mm Hg), Log urinary-albumin-to-creatinine ratio (−0.32) and eGFR (−6.4 mL/min/1.73 m^2^), and increased the frequency of serum potassium >5.5 mmol/L (4.8% vs. 0.9%)

ACD, all-cause death; ACEI, angiotensin converting enzyme inhibitor; AEs, adverse events; AF, atrial fibrillation; AMI, acute myocardial infarction; ApoB, apolipoprotein B; ARB, angiotensin receptor blocker; ASCVD, atherosclerotic cardiovascular disease; ATTR-CM, transthyretin amyloid cardiomyopathy; BARC, Bleeding Academic Research Consortium; BMI, body mass index; BW, body weight; CAD, coronary artery disease; CI, confidence interval; CKD, chronic kidney disease; CRP, C-reactive protein; CV, cardiovascular; DAPT, dual antiplatelet therapy; DB, double-blind; DD, double-dummy; DOACs, direct oral anticoagulants; FU, follow-up; eGFR, estimated glomerular filtration rate; GI, gastrointestinal; HbA1C, glycated haemoglobin; HDL-C, high-density lipoprotein cholesterol; HR, hazard ratio; HF, heart failure; HFrEF/HFmrEF/HFpEF, heart failure with reduced/mildly reduced/preserved ejection fraction; HoFH, homozygous familial hypercholesterolaemia; HR, hazard ratio; ICH, intracranial haemorrhage; IV, intravenous; KCCQ-CSS/OS, Kansas City Cardiomyopathy Questionnaire-Clinical Summary Score/Overall Summary; LDL-C, low-density lipoprotein cholesterol; LV, left ventricular; LVEF, left ventricular ejection fraction; LVOT-G, left ventricular outflow tract gradient; MACE, major cardiovascular event; MI, myocardial infarction; mRS, modified Rankin scale; 6-MWD, 6-minute walk distance; -proBNP, N-terminal pro-B-type natriuretic peptide; NYHA, New York Heart Association., once daily; OL, open label; PAD, peripheral artery disease; PAH, pulmonary arterial hypertension; PC, placebo-controlled; PCSK9, proprotein convertase subtilisin/kexin type 9; pVO2, peak oxygen consumption; QoL, quality of life; R, randomized; RR, risk reduction; SAE, serious adverse event; SC, subcutaneous; T2D, type 2 diabetes mellitus; TG, triglyceride; uCAR, urine albumin-to-creatinine ratio; VTE, venous thromboembolism; WHO, World Health Organization.

^a^In alphabetic order.

^b^Acronyms of the trials are summarized in Supplementary material online, *Table S7*.

**Table 2:** New drugs in phase 2 and 3 of clinical development^a^

Pharmacological class	Drug	Mechanism of action
Antihypertensive drugs	Baxdrostat	Highly selective aldosterone synthase inhibitors
	Lorundrostat	
	Vicadrostat	
	Zilebesiran	siRNA targeting hepatic *ANG* mRNA expression
Antiarrhythmics	CRD-4730	CaMKII inhibitor
Anticoagulants (Fact XIa inhibitors)	Abelacimw	mAb that binds to the catalytic domain of FXI
	Asundexian	Small molecule, orally active
	Milvexian	Small molecule, orally active
	REGN9933	mAb that binds and inhibits FXIa activation
Antiobesity drugs	CagriSema	Combination of cagrilintide, a long-acting amylin analogue, with semaglutide
	Maridebart cafraglutide	Peptide–antibody conjugate acting as GLP-1 receptor agonist and GIP receptor antagonist
	Mazdutide	GLP-1 and glucagon receptor dual agonist
	Orforglipron	GLP-1 receptor agonist
	Pemvidutide	GLP-1 and glucagon receptor dual agonist
	Retatrutride	Agonist of GIP, GLP-1, and glucagon receptors
	Survodutide	GLP-1 and glucagon receptor agonist
Antiplatelets	Glenzocimab	Humanized Fab fragment 9O12 against the extracellular domains of GPVI
	Ralinepag	Selective prostacyclin receptor agonist
	Selatogrel	P2Y12 receptor antagonist
Dyslipidaemia	DR10624	Fc fusion protein tri-agonist of GLP-1, glucagon and FGF21 receptors
	Enlicitide decanoate	Oral PCSK9 inhibitor
	Laroprovstat	Small molecule inhibitor of PCSK9
	Lepodisiran	GalNAC-conjugated siRNA that inhibits the hepatic synthesis of Lp(a)
	Lerodalcibep	Recombinant fusion protein of a PCSK9-binding domain (adnectin) and human serum albumin
	Muvalaplin	Oral small molecule that inhibits the apo(a)-apo B_100_ interaction
	Obicetrapib	Cholesteryl ester transfer protein inhibitor
	Olezarsen	Ligand conjugated ASO targeting apoC-III
	Olpasiran	siRNA that prevents assembly of Lp(a)
	Pelacarsen	ASO targeting the mRNA transcribed from the *LPA* gene
	Plozasiran	siRNA targeting the hepatic production of apo C-III
	Recaticimab	PCSK9 inhibitor
	SHR-1918	Fully humanized mAb against ANGPTL-3
	Tafolecimab	Fully human IgG2 mAb that specifically binds to PCSK9
	Zerlasiran	siRNA to inhibit Lp(a) production
	Zodasiran	siRNA targeting *ANGPTL3* gene expression
ET-1R antagonist	Zibotentan	Selective ET-A receptor antagonist
Fabri disease	Lucerastat	Glucosylceramide synthase inhibitor
	Migalastat	Chaperone that stabilizes misfolded, unstable α-galactosidase A enzymes
	Venglustat	Glucosylceramide synthase inhibitor
Friedreich’s cardiomyopathy	LX2006	AAV-based gene therapy designed to deliver a functional frataxin-*FXN* gene
Hypertrophic cardiomyopathy	Aficamten	Cardiac myosin inhibitor
	BMS-986435	Cardiac myosin modulator
	Ninerafaxstat	Partial fatty acid oxidation (pFOX) inhibitor
	TN-201	AVV9-based gene therapy designed to deliver the *MYBPC3* gene
Heart failure	Balcinrenone	MR modulator with partial antagonist activity
	CDR132L	Specific ASO, miR-132 inhibitor
	Ziltivekimab	Human mAb targeting the interleukin-6 ligand
Pulmonary hypertension	Sotatercept	Recombinant activin receptor type IIA-Fc (ActRIIA-Fc) fusion protein
Systemic amyloidosis	AT-02 (zamu- bafusp alfa)	Immunoglobulin-peptide fusion protein comprising a humanized IgG1 and a pan-amyloid reactive peptide, p5R
Transthyretin-mediated amyloid cardiomyopathy	ALXN2220	Recombinant human anti-ATTR IgG1 mAb
	Coramitug	Humanized mAb designed to target and clear the non-native transthyretin aggregates
	Eplontersen	Ligand-conjugated ASO to inhibit the production of hepatic TTR
	NTLA-2001	Knocking out the *TTR* gene

AAV9, adeno-associated virus serotype 9; *ANG*: gene encoding angiotensinogen; *ANGPTL3*: gene encoding angiopoietin-like protein 3; Apo, apolipoprotein; ASO, antisense oligonucleotide; CaMKII, calcium/calmodulin dependent protein kinase II inhibitor; ET-A; entothelin-1 type A receptor; Fab, Fragment antigen-binding; FGF21, fibroblast growth factor 21; FXI, coagulation factor XI; GalNAc, *N*-acetyl-galactosamine; GIP, glucose-dependent insulinotropic polypeptide; GLP1, glucagon-like peptide 1; GPVI, glycoprotein IV; Ig, immunoglobulin; Lp(a), lipoprotein (a); *LPA*, lipoprotein(a) gene; mAb, monoclonal antibody; MR, mineralocorticoid receptor; mRNA, messenger RNA; *MYBPC3*, gene encoding myosin binding protein C3; PCSK9, proprotein convertase subtilisin/kexin type 9; RNA, ribonucleic acid; siRNA, small-interfering RNA.

^a^The National Clinical Trial (NCT) number and acronyms of the ongoing clinical trials are shown in Supplementary material online, *Tables S6* and *S7*, respectively.

## New drugs approved in 2025

Compared with previous years, 2025 could be described as extraordinary, as four new CV drugs were approved.

### Aficamten

This is a selective, allosteric cardiac myosin-inhibitor that is approved to treat cardiac hypercontractility in patients with symptomatic obstructive hypertrophic cardiomyopathy (oHCM) by reducing actin-myosin cross-bridge formation. Compared with mavacamten, it has a rapid onset of action (peak plasma levels in 1.5–2 h) and a shorter half-life (75–85 h) allowing more rapid dose escalation and adjustment. Since it is metabolized by several cytochrome P450-enzymes (CYP2C9, CYP2D6, and CYP3A) clinically-relevant drug–drug interactions due to exposure to other CYP-inhibitory drugs or presence of genetically-determined poor metabolism are not expected.^26^ The approval was based on the results from the SEQUOIA-HCM trial showing that at 24 weeks, aficamten significantly improved peak oxygen uptake (pVO_2_), Kansas City Cardiomyopathy Questionnaire-Clinical Summary Scores (KCCQ-CSS) and NYHA functional class, reduced N-terminal pro-B-type natriuretic peptide (NT-proBNP) and high-sensitivity cardiac troponin I levels and led to a 50 mmHg greater reduction in Valsalva left ventricular (LV) outflow tract (LVOT) gradient (49.3% with aficamten vs. 3.6% with placebo).^27^ The aficamten group had 78 fewer days eligible for invasive septal reduction (all *P* < 0.0001). These effects were consistent across all prespecified subgroups, including patients receiving β-blockers. The incidence of AEs appeared to be similar in the two groups.

### Etripamil

Is a self-administered fast-acting (peak plasma-levels within 7 min; terminal half-life 2.5 h)^28^ verapamil analogue that prolongs refractoriness and slowing conduction through the atrioventricular node. It was approved for rapid conversion of acute symptomatic episodes of paroxysmal supraventricular tachycardia (PSVT) to sinus rhythm. It the RAPID trial recruiting 692 patients, PSVT patients self-administered a first dose of intranasal etripamil (70 mg) and a second dose if symptoms persisted 10 min after the first dose. Conversion rates at 30 min were 64.3% with etripamil vs. 31.2% with placebo (HR 2.62; 1.66–4.15; *P* < 0.0001); median time to conversion was 17.2 min with etripamil and 53.5 min, with placebo.^29^ The most frequent AEs were nasal discomfort, nasal congestion, and rhinorrhoea, but no serious AEs or deaths were reported.

### Lerodalcibep

A small recombinant fusion protein of a PCSK9-binding domain (adnectin) and human serum albumin inhibits the binding of PCSK9 to low-density lipoprotein (LDL) receptors, preventing their degradation thereby lowering LDL-cholesterol (LDL-C). The approval was based on the results of the 52-week LIBerate-HR trial, where lerodalcibep dosed monthly reduced LDL-C (by 60.3%) in patients with risk of atherosclerotic CVD (ASCVD),^30^ and the LIBerate-HeFH trial involving patients with heterozygous familial hypercholesterolaemia requiring additional LDL-C lowering, where at 24 weeks lerodalcibep reduced LDL-C by 58.6%, with 68% of participants achieving a LDL-C reduction ≥50%.^31^ Lerodalcibep-related AEs were similar to placebo. Lerodalcibep has been approved to reduce LDL-C in adults with hypercholesterolaemia, including HeFH.

### Plozasiran

**Figure 1:**
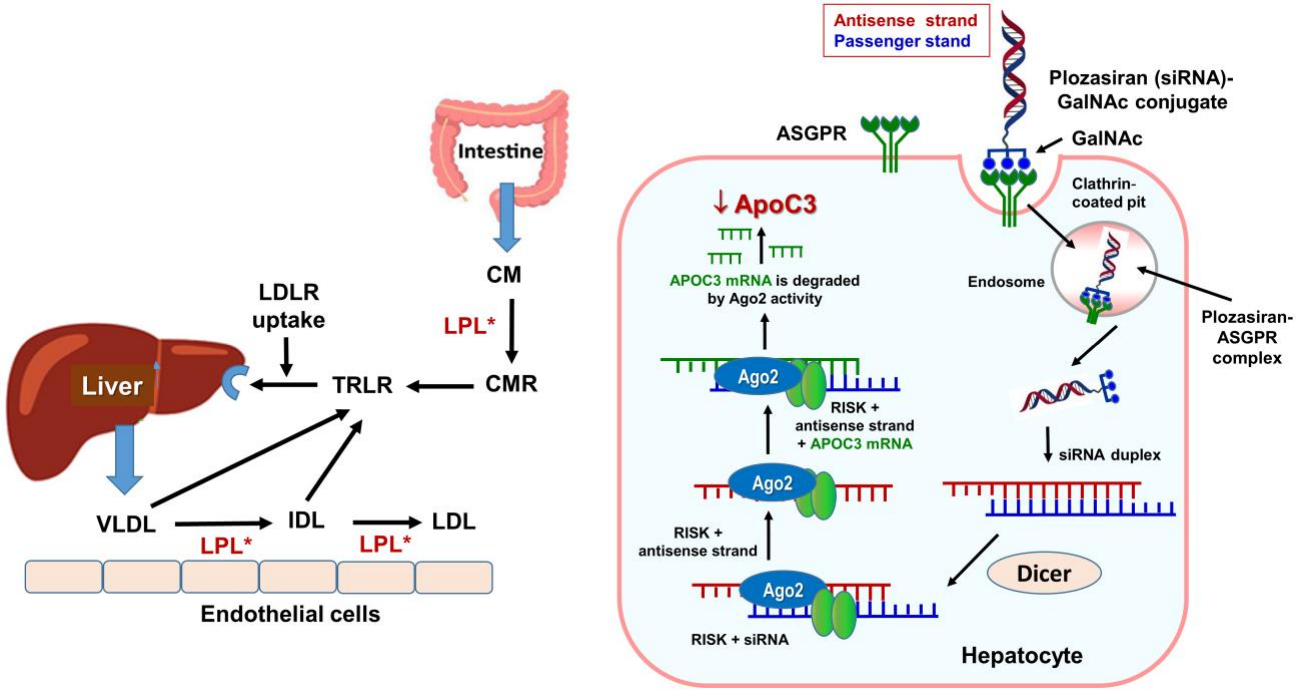
(*A*) Role of lipoprotein lipase (LPL*) in triglyceride and (*B*) mechanism of action of plozasiran (ARO-APOC3). (*A*) ApoC3 is a protein synthesized in the liver and mainly located at the surface of triglyceride-rich lipoproteins, i.e. chylomicrons (QM) generated by the enterocytes in the small intestine and very-low-density lipoproteins synthesized and released from the liver. ApoC3 inhibits the activity of lipoprotein lipase, an enzyme anchored to the luminal surface of the capillary endothelium in skeletal muscle and adipose tissue. This enzyme hydrolyzes triglyceride in the core of the particle, releasing free fatty acids and glycerol into the underlying tissue bed, leading to the formation of intermediate-density lipoprotein cholesterol, low-density lipoprotein cholesterol, and chylomicron remnants (QMR), which bind to hepatic low-density lipoprotein receptors, clearing them from the circulation. (*B*) Plozasiran is an ApoC3-directed small-interfering ribonucleic acid that specifically targets and degrades the messenger RNA encoding ApoC3. Plozasiran contains a covalently linked ligand containing three N-acetylgalactosamine residues that bind to the asialoglycoprotein receptor that is predominantly expressed on the surface of the hepatocytes, resulting in rapid endocytosis. Once in the cytoplasm, the long double-stranded RNA is recognized and cleaved by an endo-ribonuclease (Dicer) into siRNA fragments that are then incorporated into other proteins to form the RNA-induced silencing complex, and the passenger strand is removed. The siRNA-RNA-induced silencing complex containing the antisense strand [specifically the Argonaute-2 endonuclease (Ago2) within RNA-induced silencing complex] can now scan and bind to the APOC3 mRNA, producing its cleavage. By degrading apoC-III mRNA in the hepatocyte, plozasiran reduces the hepatic and serum ApoC3 levels, leading to an increased breakdown and clearance of triglyceride-rich lipoproteins. Thus, plozasiran has been approved to reduce triglyceride levels in adults with familial chylomicronaemia syndrome.

A small-interfering ribonucleic acid (siRNA) targeting hepatic apolipoprotein-C-III (apoC-III) messenger ribonucleic acid (mRNA) represents the first-approved siRNA for treatment of familial chylomicronaemia syndrome. ApoC-III regulates triglyceride-metabolism by inhibiting lipoprotein-lipase, thus delaying the clearance of triglyceride-rich lipoproteins (TRLs) (*Figure 1*). By silencing the *APOC3*-gene, plozasiran leads to more effective TRL clearance, reducing circulating triglycerides. Approval was based on PALISADE study (*Table 1*), which found that in patients with persistent chylomicronaemia or severe hypertriglyceridaemia, plozasiran (25 or 50 mg) reduced from fasting triglycerides by −80% and −78% and apoC-III by −93% and −96%, respectively vs. placebo (all *P* < 0.001). At 56 weeks follow-up, episodes of acute pancreatitis occurred in 5% of patients receiving plozasiran, and in 20% on placebo (odds ratio 0.17; 0.03–0.94; *P* = 0.03).^18^ The most common AEs were abdominal pain, nasopharyngitis, headache nausea, and hyperglycaemia. Based on these results, only 25 mg were approved.

## Drugs with label extension in 2025

Four drugs have received an extension of their primary indications.

### Oral semaglutide

The SOUL trial showed that in patients with type-2 diabetes (T2D), ASCVD, and/or chronic kidney disease (CKD), oral semaglutide, a glucagon-like peptide-1 (GLP-1) receptor agonist, at 4 years reduced the risk of major CV events (MACE), a composite of CV-related death, myocardial infarction (MI), and stroke, vs. placebo (0.86; *P* = 0.006; *Table 1*).^11^ Oral semaglutide was approved to reduce the risk of MACE in adults with T2D.

### Finerenone

The FINEARTS-HF study found that in adults with heart failure (HF) and a LV ejection-fraction (LVEF) ≥ 40%, i.e. with mildly reduced (HFmrEF) or preserved EF (HFpEF), finerenone, a nonsteroidal mineralocorticoid receptor-antagonist, reduced the rate of a composite of total worsening HF-events and CV-death vs. placebo (0.84; *P* = 0.007).^32^ Finerenone was approved to reduce the risk of CV-death, HF-hospitalization, and urgent HF visits in patients with HFmrEF or HFpEF.

### Vutrisiran

The HELIOS-B trial revealed that in patients with wild-type or hereditary transthyretin (TTR) amyloidosis with cardiomyopathy (ATTR-CM), vutrisiran, an siRNA that inhibits hepatic production of TTR, reduced the risk of all-cause death and recurrent CV-events vs. placebo (0.72; *P* = 0.01) and preserved functional capacity and quality-of-life (using the KCCQ) in patients with or without tafamidis treatment.^8^ Adverse effects similarly occurred in the vutrisiran and placebo groups. Vutrisiran was approved for the treatment of wild-type or hereditary ATTR-CM.

### Eplontersen

The NEURO-TTRansform trial showed that eplontersen, an antisense oligonucleotide (ASO) targeting TTR mRNA that inhibits the hepatic production of TTR, significantly lowered serum TTR by 80% and improved neuropathy impairment and quality of life.^33^ Eplontersen was approved for the treatment of hereditary TTR-mediated amyloidosis with stage-1/-2 polyneuropathy.

### Tirzepatide

The SURMOUNT-OSA trial found that in patients with moderate-severe obstructive sleep apnoea (OSA) and obesity, tirzepatide, a dual GLP-1 and glucose-dependent insulinotropic polypeptide (GIP) receptor agonist, reduced the apnoea–hypopnoea index, body weight and hypoxic burden, and improved sleep-related patient-reported outcomes.^34^ Thus, tirzepatide is the first drug approved for moderate-severe OSA in adults with obesity.

## Clinical trials with positive results

The phase 3 RCTs with positive outcome are summarized in *Table 1*. Results from phase 4 trials and *post hoc* analyses are summarized in Supplementary material online, *Tables S2* and *S3*.

### Antithrombotics

In the API-CAT trial, extended low-dose apixaban (2.5 mg bid) was non-inferior to full-dose apixaban (5 mg bid) for the prevention of recurrent venous thromboembolism (VTE) with less clinically relevant bleedings.^1^ In the **PRESTIGE-AF** trial, DOACs effectively prevented ischaemic strokes and other major ischaemic adverse outcomes in survivors of intracerebral haemorrhage with AF but also increased the risk of recurrent intracerebral haemorrhage and other major bleeding complications, without differences in mortality.^2^ Thus, the benefits and risks of anticoagulation in these vulnerable patients should be carefully balanced.

In the ASSET-IT trial, early tirofiban administration within 1-h post-thrombolysis in patients with acute ischaemic non-cardioembolic stroke increased the likelihood of better functional outcome (a score of 0 or 1 on the modified Rankin scale, mRS) at 90 days vs. placebo, with a higher risk of symptomatic intracranial haemorrhage (1.7% vs. 0%) and no differences in mortality.^3^

In the TARGET-FIRST trial, P2Y12-inhibitor monotherapy was non-inferior to continued DAPT (including aspirin) with respect to primary outcome (composite of ACD, MI, stent thrombosis, stroke, or BARC Type 3 or 5 bleeding), but resulted in a lower incidence of bleedings (BARC Type 2, 3, or 5) at 1-year follow-up.^4^

### Fibrinolytics

In the BRIDGE-TNK trial, functional independence at 90 days (primary endpoint) was higher with IV tenecteplase plus endovascular therapy (EVT) than with EVT alone (*P* = 0.04),^5^ with no between-group differences in ICH within 48 h and mortality at 90 days. The EXPECTS including patients with posterior circulation stroke, at 90 days, a higher percentage of patients in the alteplase group had functional independence (*P* = 0.01) or no disability than in the standard-treatment group, with no differences in ICH or mortality between groups.^6^

### Beta-blockers

In the GErman Italian Spanish Takotsubo (GEIST) Registry, during a mean follow-up of 2.6 years, β-blockers reduced mortality of patients with Takotsubo syndrome (0.71; 0.55–0.90; *P* = 0.006),^7^ with no effect on recurrence or LVEF recovery.

### Cardiomyopathies

The MAPLE-HCM trial compared aficamten with metoprolol in patients with symptomatic oHCM. At 24 weeks, aficamten was superior to metoprolol monotherapy in improving peak oxygen uptake (pVO_2_) (*P* < 0.001) and decreased symptoms, NYHA class, LV outflow tract gradient (LVOT-G), left atrial volume index, NT-proBNP levels, and KCCQ-CSS (all *P* < 0.01).^9^ However, metoprolol did not improve LVOT-G gradient, NT-proBNP levels, or left atrial volume index without differences in LV mass index or AEs between the two groups. Of note, by design, in this trial, the enrolled patients had less-severe disease burden than in other recent RCTs.

### Digitalis

In the DIGIT-HF study, 36-month treatment with digitoxin (titrated to a serum concentration of 8–18 ng/mL) significantly reduced the risk of ASCVD or hospitalization for worsening HF in patients with HFrEF among all prespecified subgroups.^12^ However, digitoxin is not available in many countries.^10^

### Incretin-based therapies

The STRIDE trial investigated the effect of semaglutide in patients with peripheral artery disease (PAD) and T2D. At week 52, semaglutide significantly improved the median difference in 6-MWD, pain-free walking distance, and quality of life as compared with placebo.^12^ This benefit was apparent already at 26 weeks and continued over time. The SUMMIT trial showed that in patients with HFpEF and a body mass index ≥ 30, 2 years treatment with tirzepatide of reduced the composite of CV-death or worsening HF (0.62; *P* = 0.026), and decreased body weight and high-sensitivity C-reactive protein, blood pressure, and improved health status (based on CCQ-CSS) and 6-MWD (*P* < 0.001 vs. placebo).^13^ The effect of tirzepatide was consistent in all subgroups and was associated with similarly decreased LV-mass and visceral adiposity (paracardiac fat) in patients with and without T2D.^35^ Of note, absolute risk reduction in primary events was numerically greater in patients with CKD.^36^ The SURMOUNT-5 trial compared tirzepatide and semaglutide obese patients without T2D.^14^ At 72 weeks, body weight decreased by 20.2% and 13.7%, waist circumference decreased by 18.4 cm and 13.0 cm, with tirzepatide and semaglutide, respectively (*P* < 0.001 for each). Thus, tirzepatide was superior to semaglutide in the reduction of body weight.

### Lipid-lowering drugs

The VESALIUS-CV trial showed that patients with atherosclerosis or diabetes without a previous MI or stroke, the PCSK9-inhibitor evolocumab added on top of lipid-lowering therapy significantly reduced both 3-point (−25%) and 4-point MACE (19%) and the composite of CV-death, MI, or ischaemic stroke (−21%), death from CAD or MI (−27%), MI (−36%), and ischaemia-driven arterial revascularization (−21%) without increasing the incidence of AEs (*P* < 0.001 for all).^19^ These results confirmed the benefits of intensive LDL-C lowering in high-risk patients irrespective of prior events. In the ORION-13 trial patients with homozygous familial hypercholesterolaemia 1-year treatment with inclisiran on top of maximum tolerated doses of statins significantly reduced LDL-C by 33.3%, with a corresponding reduction in PCSK9 activity of 60.2%,^17^ supporting the efficacy of inclisiran on a statin background. Among patients with severe hypertriglyceridaemia (fasting triglycerides-TG ≥500 mg/dL), 6-month treatment with the selective ApoC-III inhibitor olezarsen (50-mg or 80-mg) reduced the placebo-adjusted triglyceride levels by 62.9% and 72.2% in the CORE-TIMI 72a trial, and by 49.2% and 54.5% in the CORE2-TIMI 72b trial, respectively; all *P* < 0.001 vs. placebo.^15^ Olezarsen also decreases the levels of ApoC3, remnant cholesterol, and non-HDL cholesterol and the incidence of acute pancreatitis compared with placebo. The ESSENCE TIMI73b trial evaluated the efficacy of olezarsen in patients with moderate hypertriglyceridaemia (100–400 mg/dL) and high CV risk or with severe hypertriglyceridaemia. At 6 months, olezarsen (50 mg or 80 mg) reduced the placebo-adjusted triglycerides levels by 58.4% and 60.6%, in patients with severe hypertriglyceridaemia, and by 85.0% and 88.7% of patients moderate hypertriglyceridaemia, respectively (*P* < 0.001 for both).^16^ The incidence of severe AEs was similar across trial groups.

### Drugs against pulmonary hypertension

The ZENITH trial evaluated the efficacy and safety of sotatercept, an activin-signalling inhibitor, in patients with pulmonary arterial hypertension (PAH, WHO functional class III-IV), a high 1-year risk of death and a REVEAL Lite 2 risk score ≥9 who were receiving maximum tolerated doses of background therapy.^21^ The trial was stopped early based on the efficacy results of a prespecified interim analysis, which found that sotatercept significantly reduced the risk of a composite of ACD, lung transplantation, or hospitalization (≥24 h) for worsening PAH as compared with placebo (0.24; 0.13–0.43; *P* < 0.001). The HYPERION trial assessed the effects of early initiation of sotatercept in patients with WHO functional class II-III PAH who had received the diagnosis <1 year earlier, had intermediate-high risk of death, and were receiving double or triple background therapy. Sotatercept resulted in a lower risk of clinical worsening (composite of ACD, unplanned hospitalization lasting at least 24 h for worsening of PAH, atrial septostomy, lung transplantation, or deterioration in performance in exercise testing due to PAH) than placebo (*P* < 0.001), an effect consistent across all prespecified subgroups.^20^ The early and sustained separation of Kaplan–Meier curves for the primary endpoint indicated that a clinical benefit could be detected after three doses of sotatercept. Based on this result, the steering committee decided to stop this trial.

### Non-steroidal mineralocorticoid receptor antagonists

The CONFIDENCE trial studied the combination of finerenone and empagliflozin in patients with CKD and T2D receiving maximum tolerated doses of a RAASi. After 180 days, finerenone plus empagliflozin led to a greater reduction in the urine albumin-to-creatinine ratio than either drug alone (52%), suggesting an additive effect on the basis of the reductions observed with finerenone alone (32%) and empagliflozin alone (29%; *P* < 0.001 for both).^22^ Incidence of hyperkalaemia was 15%–20% lower with combination therapy as compared with finerenone alone, and a decrease in the eGFR >30% at day 30 occurred in 6.3%, 3.8%, and 1.1% of patients in the combination, finerenone, and empagliflozin groups, respectively. Neither agent, alone or in combination, led to unexpected AEs.

### Sodium-glucose transporter type 2 inhibitors

The DECODE-CKD trial including patients with CKD, treatment with dapagliflozin for 6 months significantly reduced LV mass index compared with placebo (−8.44 g/m^2^; *P* < 0.001), an effect across prespecified subgroups. The rate of serious adverse events was similar between groups.^23^ The SOGALDI-PEF trial compared the efficacy and safety of dapagliflozin–spironolactone combination vs. dapagliflozin alone in patients with CKD.^25^ Compared with dapagliflozin, dapagliflozin/spironolactone combination reduced *Log*NT-proBNP levels (*P* = 0.035) and more patients in the combination therapy group reached NT-proBNP reduction ≥20% (*P* = 0.016). Combination therapy reduced SBP, BW, Log urinary-albumin-to-creatinine ratio and eGFR but increased the risk of hyperkalaemia. The 2-year post-trial follow-up of the EMPA-KIDNEY study was designed to assess whether the cardio-renal benefits of empagliflozin would persist after the drug discontinuation.^24^ No study drug was issued during the post-trial period, but local practitioners could prescribe open-label sodium-glucose transporter type 2 inhibitor, including empagliflozin. During the post-trial period only, empagliflozin reduced primary composite outcome of kidney disease progression or CV death (0.87; 0.76–0.99) and during the combined active- and post-trial periods, empagliflozin reduced the progression of kidney disease or CV death (0.79; 0.72–0.87) but not non-CV death. Thus, the cardiorenal benefits of empagliflozin persist for up to 12 months after drug discontinuation.

## Clinical trials with neutral or negative outcome

The characteristics and main results of these trials are shown in Supplementary material online, *Table S4*.

### Antithrombotics

The DOAC-CVT trial aimed to compare DOACs and vitamin K antagonists (VKAs) in patients with cerebral vein thrombosis starting OAT within 30-days after diagnosis.^37^ At 6-month follow-up, the rate of recurrent thrombosis and major bleeding did not differ between patients treated with DOACs vs. VKAs. The HOST-BR trial studied the optimal DAPT duration in patients at high-(HBR) and low-bleeding risk (LBR) who underwent PCI with a drug-eluting stent.^38^ At 12-months, in HBR patients, those who received 3-month DAPT had less net-adverse clinical events and MACCE than those who received 1-month, without differences in bleeding In LBR patients, 3-month DAPT was non-inferior to 12-month DAPT regarding net-adverse clinical events and MACCE but caused less bleeding (*P* < 0.0001). In the NEO-MINDSET non-inferiority trial,^39^ monotherapy with a potent P2Y12-inhibitor (ticagrelor or prasugrel) at 12-months was not inferior to DAPT with aspirin and ticagrelor or prasugrel with regard to a composite of ACD, MI, stroke, or urgent target-vessel revascularization. Haemorrhagic events were observed somewhat less frequently in the monotherapy group than in the DAPT group. The OCEAN trial reported that in patients with risk factors for stroke who had undergone successful catheter ablation for AF, rivaroxaban did not reduce the incidence of stroke, systemic embolism, or new covert embolic stroke than treatment with aspirin.^40^ The incidence of fatal or major bleeding appeared to be similar in the two groups, whereas minor bleeding and clinically relevant non-major bleeding were higher in the rivaroxaban group. The START trial determined the optimal time-to-initiate anticoagulation with a DOAC after ischaemic stroke in patients with non-valvular AF. Although an optimal time point to initiate DOAC within the first 14-days was not identified, the use of response-adaptive randomization suggested that initiation of DOAC was better than later time points post-stroke.^41^

The TADCLOT trial tested the superiority of ticagrelor vs. clopidogrel (75 mg bid) in reducing death, MI, stent thrombosis, stroke, or target lesion revascularization in patients with STEMI within the first month post-primary PCI.^42^ At 30-days, the primary endpoint occurred in 2.2% vs. 2.9% in ticagrelor and twice-daily clopidogrel, respectively (*P* = 0.28). Thus, ticagrelor was not superior to clopidogrel in reducing MACE within the first 1-month post-primary PCI. Among high-risk patients undergoing complex PCI, the tailored TAILORED-CHIP trial found that antiplatelet strategy with early escalation and late de-escalation, as compared with DAPT, did not decrease the incidence of primary net adverse-events (composite of ACD, MI, stroke, stent thrombosis, unplanned urgent revascularization, or clinically relevant bleeding) at-12 months, compared with standard DAPT.^43^ However, clinically relevant bleeding was higher in the tailored therapy group than in the DAPT group (*P* = 0.002).

### Colchicine

The CLEAR trial showed that colchicine started soon after MI did not reduce the composite primary outcome of CV death, recurrent MI, stroke, or unplanned ischaemia-driven coronary revascularization vs. placebo.^44^ In the COCOMO-ACS trial recruiting patients with non-ST-segment elevation MI (NSTEMI), colchicine had no effect on the minimum fibrous cap thickness or maximum lipid arc non-culprit segments; the small size and short follow-up (17 months) are major limitations of this study.^45^

### Diuretics

Among patients with NSTEMI who had undergone PCI, the CLEAR trial found that spironolactone did not reduce the composite of CV death or new or worsening HF or the incidence of a composite of CV death, MI, stroke, or new or worsening HF.^46^

### Glucose-lowering drugs

In patients with PAD without diabetes, the PERMET trial reported that 6-month treatment with metformin did not improve the 6-MWD distance as compared with placebo (−5.4 m vs. −5.3 m).^47^

### Heart failure

The FAIR-HF2 DZHK05 showed that in patients with HFrEF and iron deficiency, IV ferric carboxymaltose did not reduce the time to first HF hospitalization or CV death in the overall cohort or in patients with a transferrin saturation <20% or reduce the total number of HF hospitalizations vs. placebo.^48^ The REBOOT-CNIC trial evaluated the effect of β-blocker therapy in patients with AMI that received invasive care during the index hospitalization and a LVEF >40% before discharge. At 3.7 years, β-blocker therapy was not associated with a lower incidence of ACD, reinfarction, or HF hospitalization, with no between-group differences in safety outcomes.^49^ A meta-analysis of five contemporary trials reported that β-blockers did not reduce the incidence of death of any cause, MI, or HF in patients with a recent MI and preserved LVEF (≥50%) without other indications for β-blocker therapy.^50^ The VICTOR trial showed that in ambulatory patients with chronic HFrEF without recent HF worsening, vericiguat did not reduce the risk of CV-death or HF hospitalization or time-to-first HF hospitalization.^51^ However, in a pooled patient-level analysis of the VICTORIA (recruiting patients with reduced LVEF and recent HF decompensation) and VICTOR trials including 11 155 participants with HFrEF, vericiguat reduced the risk of CV-death or HF hospitalization vs. placebo (HR 0.91; *P* = 0.008), with similar reductions in each individual component.^52^ These findings suggest that vericiguat might be a new therapeutic option for patients with HFrEF.

### Lipid-lowering drugs

The CAVIAR trial found that in patients with low LDL-C levels, the addition of alirocumab to statin therapy for 1 year early after heart transplantation safely reduced mean LDL-C levels (from 72 to 31 mg/dL; *P* < 0.001) but did not reduce coronary artery plaque progression compared with rosuvastatin.^53^ Furthermore, the NEWTON-CABG trial found that early, intensive LDL-C lowering with evolocumab on top of statins did not significantly reduce 24-month saphenous-vein graft (SVG) disease, despite achieving a ∼48% greater reduction in LDL-C compared with placebo, suggesting that graft thrombosis rather than atherosclerosis may be the main mechanism of early-graft failure.^54^

### Mavacamten

The ODYSSEY-HMC trial reported that patients with symptomatic non-obstructive HCM (noHCM), mavacamten did not improve exercise capacity (measured by pVO_2_) or decreased symptoms (assessed by the KCCQ-CSS) or NT-proBNP levels compared with placebo.^55^ Reductions in LVEF (<50% or ≤30%), AF, and permanent interruptions in drug treatment were more common with mavacamten than with placebo.

### Sodium-glucose transporter type 2 inhibitor

The DAPA ACT HF-TIMI 68 trial evaluated the efficacy and safety of in-hospital initiation of dapagliflozin in HF-hospitalized patients. After 2 months, dapagliflozin did not reduce the risk of CV-death, worsening HF or ACD but increases the rates of symptomatic hypotension and worsening kidney function.^56^ However, a prespecified meta-analysis of this trial and two other trials suggested that in-hospital initiation of SGLT2i may reduce the risk of early CV death or worsening HF and ACD.

## Withdrawal of CV drugs

Andexanet-alfa, a recombinant modified human ‘decoy’ factor Xa (FXa) protein, was approved in 2018 to manage life-threatening or uncontrolled bleeding associated to rivaroxaban or apixaban based on a surrogate endpoint (the change from baseline in anti-FXa activity in healthy volunteers). However, in the ANEXA-I trial, Andexanet-alfa increased the incidence of thrombosis (14.6% vs. 6.9%) and thrombosis-related deaths at day 30 (2.5% vs. 0.9%) as compared with usual care (https://www.fda.gov/safety/medical-product-safety-information/update-safety-andexxa-astrazeneca-fda-safety-communication). Therefore, Andexanet-alfa was pulled from the US market.

## Cardiovascular drugs under clinical development

Multiple drugs with different mechanisms of action are currently evaluated in phase 2 (mainly dose-finding) and phase 3 RCTs that analyse their efficacy and safety in the prophylaxis and treatment of a variety of CVD. Phase 2 and 3 studies published in 2025 with drugs under clinical development are summarized in Supplementary material online, *Table S5*, while ongoing phase 2 and 3 clinical trials with drugs under clinical development are summarized in Supplementary material online, *Table S6*. Below, we briefly summarize the most relevant drug developments.

### Antihypertensive drugs

The treatment of uncontrolled and resistant hypertension often requires administration of 2–5 antihypertensive drugs. Zilebesiran is a siRNA conjugated to a triantennary N-acetylgalactosamine (GalNAc) ligand to facilitate its specific uptake by asialoglycoprotein receptors located on hepatocytes where zilebesiran selectively inhibits hepatic angiotensinogen. Due to its high-metabolic stability and sustained intracellular activity, zilebesiran is administered subcutaneously every 3–6 months.^57^ Specific aldosterone synthase (CYP11B2) inhibitors like baxdrostat, lorundrostat, or vicadrostat constitute another promising antihypertensive option. Both strategies represent complementary upstream and downstream approaches that inhibit the renin-angiotensin-aldosterone system.

### Antiobesity drugs

The treatment of patients with severe overweight and/or obesity, irrespective of presence of T2D, remains challenging. Very recently, the approval of three new GLP-1 agonists was predicted: two injectables (high-dose semaglutide 7.2 mg once-weekly, and the combination of cagrilintide, a long-acting synthetic amylin analogue, with semaglutide) and orforglipron, a daily oral, non-peptide GLP-1-receptor agonist.^58^ Other drugs with different mechanisms of action are mazdutide and survodutide, two once-weekly injectable dual agonists of GLP-1 and glucagon receptors, maridebart cafraglutide, a once-monthly injectable combining GLP-1-receptor agonism with GIP-receptor antagonism, and retatrutide, a once-weekly triple GIP/GLP-1/glucagon-receptor agonist.

### Lipid-lowering drugs

Several drugs that reduce both LDL-C and triglycerides are under development. Enlicitide decanoate, a macrocyclic peptide that inhibits the interaction between PCSK9 and LDLR, and the small-molecule inhibitor laroprovstat that binds to a novel pocket in the PCSK9 C-terminal domain without affecting the PCSK9-LDL receptor interaction are two oral PCSK9-inhibitors. Recaticimab is a fully human IgG1 monoclonal antibody engineered to achieve longer-term PCSK9 in activation. Its long half-life (18.6–27.4 days) enables dosing intervals of up to 12 weeks. RNA-based Lp(a)-lowering drugs (ASO: pelacarsen; siRNA: lepodisiran, olpasiran, and zerlasiran) silence the LPA gene responsible for the hepatic synthesis of Apo(a) and reduce Lp(a) levels by ca. 80%–95%. The orally active small molecule muvalaplin inhibits Lp(a) formation by blocking the apo(a)-apo B100 interaction and reduces Lp(a) levels by up to 65%. Drugs targeting angiopoietin-like protein 3 (ANGPTL3), a glycoprotein expressed by the hepatocytes, regulate lipid metabolism through inhibition of lipoprotein and endothelial lipases. Solbinsiran and zodasiran are two GalNAc-conjugated small siRNA-targeting hepatic ANGPTL3. They increase the activity of lipoprotein and endothelial lipases, which facilitates the breakdown of triglyceride-rich lipoproteins and reduces triglyceride and LDL-C levels in patients with mixed hyperlipidaemia or homozygous or heterozygous familial hypercholesterolaemia receiving foundational lipid-lowering therapy. Obicetrapib, a highly selective cholesteryl-ester transfer-protein inhibitor, reduces LDL-C levels in patients with either familial hypercholesterolaemia or established ASCVD receiving maximum tolerated doses of standard lipid-lowering therapy.

All above mentioned drugs under clinical development may represent new therapeutic alternatives for CVD, provided their efficacy and safety are confirmed in ongoing RCTs. Again, we witness the continued advances of biologic drugs, including ASO, siRNA, and monoclonal antibodies, with a longer half-life allowing for parenteral administration every few weeks or months, which may improve drug adherence. Of note, because they are not metabolized via hepatic cytochrome P450 isoenzymes, they carry a much lower risk of drug–drug interactions.

## Conclusions

As in previous years,^59–61^ we have summarized the most relevant advances in CV pharmacotherapy in 2025, including new drug approvals, label extensions for some already approved drugs and the results of clinical trials with positive or negative results, conducted with lipid- and glucose-lowering drugs, antiobesity medications, antithrombotic agents, and other drugs for the treatment of arterial and pulmonary hypertension, HF, and cardiomyopathies. We have also briefly presented the most promising CV drugs currently being investigated in ongoing phase 2 and 3 clinical trials. We hope that the results of these trials will confirm therapeutic efficacy and safety and lead to the approval of new, more effective, and safer pharmacological agents for the prevention and treatment of CVDs in the coming years.

## Supplementary Material

pvag016_Supplementary_Data

## Data Availability

The data underlying this review were taken from the quoted references.
