# Correction to: Cardiovascular pharmacotherapy in 2025

**DOI:** 10.1093/ehjcvp/pvag028

**Published:** 2026-04-27

**This is a correction to**: Juan Tamargo, Stefan Agewall, Giuseppe Ambrosio, Claudio Borghi, Elisabetta Cerbai, Gheorghe A Dan, Heinz Drexel, Péter Ferdinandy, Erik Lerkevang Grove, Roland Klingenberg, Joao Morais, William Parker, Bianca Rocca, Patrick Sulzgruber, Anne Grete Semb, Samuel Sossalla, Juan Carlos Kaski, Dobromir Dobrev, Cardiovascular pharmacotherapy in 2025, *European Heart Journal - Cardiovascular Pharmacotherapy*, Volume 12, Issue 3, May 2026, Pages 147–166, https://doi.org/10.1093/ehjcvp/pvag016

The following change has been made to the originally published paper. The title of the article has been corrected to read: “Cardiovascular pharmacotherapy in 2025” instead of “New pharmacological agents and novel cardiovascular pharmacotherapy strategies in 2025”.

