# Supplementary material for: Cardiovascular pharmacotherapy in 2025
Source: Eur Heart J Cardiovasc Pharmacother. 2026 Apr 5;12(3):147–66. doi: 10.1093/ehjcvp/pvag016 (PMC13185746; doi:10.1093/ehjcvp/pvag016)
Supplement: pvag016_Supplementary_Data [file pvag016_supplementary_data.docx]

**Supplemental Table 1. Search terms, data sources and strategy terms**

For the selection of the most relevant advances in CV pharmacotherapy in 2025, clinical evidence published in English up to December 2025 was searched using PubMed, Embase, Scopus, and Web of Science databases. Medical Subject Headings, data sources, and strategy terms used to effectively identify relevant information are summarized below. For the selection of the most relevant advances in CV pharmacotherapy in 2025, we reviewed individual drugs as well as fixed combinations

| **Variables** | **Inclusion** |
| --- | --- |
| Predeﬁned MESH terms | Cardiovascular drugs, antiarrhythmics, antihypertensive drugs, antiobesity drugs, antithrombotics (anticoagulants, antiplatelets, fibrinolytics), beta-blockers, diuretics, drugs for cardiomyopathies, glucose-lowering drugs, heart failure drugs, hemosthatic therapy, incretins, lipid-lowering drugs, pulmonary hypertensive drugs, renin-angiotensin aldosterone inhibitors, sodium-glucose cotransporter 2 inhibitors |
| Publications included | Clinical trials, prespecified subanalysis, post-hoc analysis  New indications and label extensions on the websites of the European Medicines Agency and the Food and Drug Administration (FDA) |
| Publications excluded | Abstracts, conference proceedings, non-placebo-controlled trials, articles in languages other than English |
| Databases | MEDLINE/PubMed, Embase, Scopus, Web of Science |
| Years of publication | Until December 2025 |
| Data originated in | Western countries |
| Selection for inclusion | Drugs and RCTs were selected for inclusion in this review based on their novelty and potential clinical impact by consensus among a group of Experts within the nucleus of the Working Group on Cardiovascular Pharmacotherapy of the European Society of Cardiology. Discrepancies regarding the inclusion/exclusion of studies were resolved by consensus. |

### **Supplemental Table 2. Phase 4 clinical trials with positive results***

| **Trial acronym**/NCT** | **Trial/Population** | **Treatment** | **Primary endpoint** | **Results (HR; 95% CI; P value)** |
| --- | --- | --- | --- | --- |
| **1. Antithrombotics** | | | | |
| **1.1. Anticoagulants** | | | | |
| ALONE-AF^1^  NCT04432220 | OL, PA trial. 840 with AF, at moderate or high stroke risk, who had undergone catheter ablation of AF, and has maintained sinus rhythm for more than 1 year. Female: 24.9%. FU: 2 years | - Discontinue or continue OAT (with DOACs) | First occurrence of a composite of stroke, systemic embolism, and major bleeding | At 2 years, discontinuing OAT reduced the risk of the primary end point vs. continuing DOAC therapy (0.3% vs. 2.2%; P =0.02). The cumulative incidence of ischemic stroke was 0.3% in the discontinue group vs 0.8% in the continue group (absolute difference −0.5%; −1.6% to 0.6%). Major bleeding: 1.4% in the continue OAT, but was not observed in the discontinue OAT group |
| ARTESIA  Subgroupp analysis^2^  [NCT01938248](https://clinicaltrials.gov/show/NCT01938248) | DB, DD, R controlled trial. 4012 with device-detected subclinical AF lasting 6 min to 24 h, a CHA2DS2-VASc score ≥3 and a history of stroke or TIA. Females: 34%. FU: 3.5 years. | Apixaban (5 mg bid or 2.5 mg bid if indicated) or aspirin 81 mg od | Composite of all-cause stroke or systemic arterial embolism, assessed as absolute risk differences | Relative to aspirin, apixaban reduced the annual rate of stroke or systemic embolism in individuals with (1.20% vs. 3.14%) or without previous stroke or TIA (0.74% vs. 1.07%; P_int_=0.24). The absolute risk reduction in stroke or systemic embolism was 7% with apixaban, which was more than double the corresponding absolute risk increase in major bleeding (3%) and nearly 7 times the absolute risk reduction in stroke or systemic embolism seen in participants without a history of stroke or TIA (1%; P_int_=0.03). The annual rate of major bleeding events at 3.5 years increased with apixaban relative to aspirin in patients with or without a history of stroke or TIA (P_int_=0.42) but apixaban did not increase the risk of fatal or ICH |
| HI-PRO^3^  NCT04168203 | DB, R trial. 600 with VTE after the occurrence of a transient provoking factor who had at least one enduring risk factor and had completed at least 3 months of OAC therapy. Female: 57%. FU: 12 months | Apixaban (2.5 mg bid) or placebo | First episode of major bleeding according to the criteria of the ISTH | Low dose apixaban reduced symptomatic recurrent VTE (1.3% vs 10.0%; HR 0.13; 0.04-0.36; P<0.001) with a low risk of major bleeding but increased clinically relevant non-major bleeding (4.8% vs. 1.7%; 2.68; 0.96-7.43; P=0.06). Non-hemorrhagic, nonfatal AEs occurred in 2% of patients in each group |
| RIVAWAR^4^  NCT04970576 | OL, noninferiority, R controlled trial. 261 diagnosed with LV thrombus within 7 days of STEMI/NSTEMI. Female: 20.7%. FU: 12 weeks | Rivaroxaban (20 mg od) or warfarin (INR 2-3) for 12 weeks | LV thrombus resolution, as assessed by echocardiography at 4 and 12 weeks | At 4 weeks, LV thrombus resolution was higher in the rivaroxaban group (20% vs 8%; *P* = 0.017), with similar resolution at 12 weeks (95.8% vs 96.6%; *P* = 0.76). There were no differences in all-cause death, ischemic stroke, or major bleeding events |
| **2.2. Antiplatelets** | | | | |
| SMART CHOICE 3^5^  [NCT04418479](http://clinicaltrials.gov/show/NCT04418479) | Investigator-initiated, P, R, OL trial. 5542 at high risk of recurrent ischemic events (previous MI, medication-treated diabetes, or complex coronary lesions) who completed a standard duration of DAPT after PCI. Female: 18%. FU: 2.3 years | Clopidogrel (75 mg od) or aspirin (100 mg od) oral monotherapy | Cumulative incidence of ACD, MI, or stroke, assessed in the intention-to-treat population | Clopidogrel reduced the cumulative incidence of MACCE compared with aspirin (4.4% vs. 6.6%; HR 0.71; 0.54-0.93; P=0.013), mainly driven by a reduction in MI (1% vs. 2.2%; 0.54; 0.33-0.90) and all-cause death (2.4% vs. 4.0%; 0.71; 0.49-1.02). There were no differences in the risk of stroke, bleeding (BARC 2, 3, or 5 types) or AEs between groups |
| **2.3. Anticoagulants vs. antiplatelets** | | | | |
| ADAPT AF-DES^6^  NCT04250116 | R, OL, non-inferiority trial. 960 with AF who had undergone the implantation  of a drug-eluting stent at least 1 year earlier. Female: 21.5%. FU: 12 months | DAOC monotherapy or combination therapy (DOAC plus clopidogrel) | Net adverse clinical events, a composite of ACD, MI, stent thrombosis, stroke, systemic embolism, or major bleeding or clinically relevant non-major bleeding | DOAC monotherapy was non-inferior to combination therapy for the primary endpoint (9.6% vs. 17.2%; 0.54; 0.37-0.77; P<0.001 for non-inferiority: P<0.001 for superiority), and of major bleeding or clinically relevant nonmajor bleeding (5.2% vs. 13.2%; HR 0.38; 0.24-0.60) |
| **2.4. Fibrinolytics** | | | | |
| ANGEL-TNK^7^  NCT05624190 | Phase 4, P, OL, blinded end point, R trial. 256 with acute large vascular occlusion treated with successful EVT between 4.5 and 24 hours of symptom onset. Females: 44% , FU: 90 days | Intra-arterial tenecteplase (IA-TNK, 0.125 mg/kg) or standard medical treatment | Excellent outcome at 90 days, defined as modified Rankin Scale (mRS) score of 0 to 1 (range, 0 [no symptoms] to 6 [death]) | A greater proportion of patients who received IA-TNK (40.5%) were disability-free (mRS 0–1) compared to the standard medical treatment (26.4%; HR 1.44; 1.06-1.95; P=0.02). There were no significant differences in sICH within 48 hours or 90-day mortality between the groups. |
| POST-TNK^8^  ChiCTR2200064809 | Investigator-initiated, R, OL, blinded outcome assessment trial. 540 with stroke due to proximal intracranial large vessel occlusion within 24 hours of the time they were last known to be well, with an eTICI score of 2c to 3 after EVT. Female: 41.4%. FU: 90 days | Intra-arterial tenecteplase (IA-TN, 0.0625 mg/kg, max 6.25 mg) vs. no IA thrombolysis | Freedom from disability, defined as a score of 0 or 1 on the mRS (range, 0 [no symptoms] to 6 [death]) at 90 days | There was no difference in excellent neurological outcomes (mRS 0-1) at 90 days (49.1% vs 44.1%, aRR 1.15; 0.97–1.36]; P=0.11), nor in symptomatic ICH or 90-day mortality (16.0% vs. 19.3%; 0.75; 0.50-1.13]; P =0.16) |
| **3. Beta-blockers** | | | | |
| BETAMI-DANBLOCK^9^  NCT03646357  NCT03778554 | OL, R trial with blinded end-point evaluation. 5574 hospitalized with MI and preserved or mildly reduced LVEF (≥40%). Female: 21%. FU: 3.5 years | Long-term β-blocker therapy (94.5% with metoprolol) within 14 days after the event or no β-blocker therapy | Composite of ACD or MACE (new MI, unplanned coronary revascularization, ischemic stroke, HF, or malignant ventricular arrhythmias) | β-blockers reduced the risk of the primary endpoint (14,2% vs. 16.3%; 0.85; 0.75-0.98; P=0.03). They may decrease the cumulative incidence of new MI (0.73; 0.59–0.92), but there were no differences in ACD, unplanned coronary revascularization, HF, malignant ventricular arrhythmias and ischemic stroke and safety outcomes between both groups |
| Subanalysis of the AβYSS trial^10^  NCT03498066 | Blinded endpoint, R, OL, noninferiority trial. 3698 patients of the AβYSS trial, with or without prior history of hypertension. Female: 17.2%. FU: 3 years | Randomized to interruption or continuation of β-blocker treatment | Changes in heart rate and BP and the impact on the primary endpoint (of all-cause death, MI, stroke, or CV hospitalizations) in the pre-specified subgroups of patients with or without prior history of hypertension | β-blocker interruption increased SBP/DBP (+3.7/+3.3 mmHg; P<0.001) and resting HR (+10 bpm; P<0.001) that persisted over the duration of follow-up. Hypertensive patients were at higher risk of events as compared with patients without hypertension (25.8% vs. 19.2%; P=0.03) and presented a marked increase in the primary endpoint (risk difference 5.02%; P=0.014) when randomized to β-blocker interruption. |
| **4. Diuretics** | | | | |
| Spironolactone vs amiloride in resistant hypertension^11^  NCT04331691 | OL, R, PA trial. 118 with resistant hypertension (SBP ≥130 mmHg after a 4-week run-in period with a fixed-dose triple medication combination) and serum potassium <5.0 mmol/L. Female: 30%. FU: 12 weeks | Spironolactone (12.5 mg up to 25 mg od) vs amiloride (5 mg up to 10 mg od) | Between-group difference in home SBP change (noninferiority margin of -4.4 mm Hg for the lower bound of the CI) | At 12 weeks, mean reduction in home SBP was -13.6 and -14.7 mm Hg and office-measured achievement rates of SBP <130 mm Hg were 57.1% and 60.3% in the amiloride and spironolactone groups, respectively. Thus, amiloride represents an alternative in patients with resistant hypertension when spironolactone is not tolerated and it may be added to low-dose spironolactone to minimize the AEs of high doses of spironolactone |
| **5. Incretin therapy** | | | | |
| OASIS 4^12^  NCT05564117 | DB, R, PC trial. 205 without T2D who had a BMI ≥30 or ≥27 with at least one obesity-related complication. Females: 80%. FU: 64 weeks | Oral semaglutide (3 mg uptitrated to 25 mg od) vs. placebo | Coprimary end points at week 64 were the percent change in BW and a reduction of in BW ≥5% | Semaglutide produced greater mean reduction in BW ≥5%, ≥10%, ≥15 or ≥20% (all P<0.001) and improved IWQOL-Lite-CT Physical Function score (16.2 vs. 8.4 points; 3.3-12.2; P<0.001) vs. placebo. GI AEs were more frequently with semaglutide than with placebo (74.0% vs. 42.2%) |
| **6. Sodium-glucose cotransporter 2 (SGLT2) inhibitors** | | | | |
| DAPA-TAVI^13^  NCT04696185 | Interventional trial. 1222 elderly (mean age 82 years) with severe aortic stenosis undergoing TAVI. All the patients had a history of HF plus at least one of the following: CKD (88.6%), T2D (44%) or HFrEF (17%). Female: 49%. FU: 1 year | Oral dapagliflozin (10 mg od) and standard care or standard care alone | Composite of all-cause death or worsening of HF, defined as hospitalization or an urgent visit, at 1 year | Dapagliflozin reduced the primary outcome vs. standard care (15.0% vs. 20.1%; 0.72; 0.55-0.95; P=0.02), due to a reduction in worsening HF (9.4% vs. 14.4%; 0.63; 0.45-0.88), but not in ACD. Genital infections (1.8% vs. 0.5%, P=0.03) and hypotension (6.6% vs. 3.6%; P=0.01) were more common in the dapagliflozin group. AEs led to dapagliflozin withdrawal in 6.1% of patients. |

***** In alphabetic order ** Acronyms of the trials are summarized in Supplemental Table 7

Abbreviations. ACD: all-cause death. AEs: adverse events. AF: atrial fibrillation. aRR: adjusted risk ratio. BARC: Bleeding Academic Research Consortium. bid: twice daily. BP: blood pressure. BW: body weight. CI: confidence interval. CKD: chronic kidney disease. DAPT: dual antiplatelet therapy. DB; double blind. DBP: diastolic blood pressure. DD: double-dummy. DOACs: direct oral anticoagulants. eTICI: expanded Thrombolysis in Cerebral Infarction. FU: follow-up. GI: gastrointestinal. HR: hazard ratio. HF: heart failure. HFrEF: heart failure with reduced ejection fraction. ISTH: International Society on Thrombosis and Haemostasis criteria. LV: left ventricular. LVEF: left ventricular ejection fraction. MACE: major cardiovascular events. MACCE: major adverse cardiovascular or cerebrovascular events. MI: myocardial infarction. mRS: modified Rankin Scale. OACT: oral anticoagulant therapy. od: once daily. OL: open label. PA: parallel assignment. PC: placebo-controlled. R: randomized. SBP: systolic blood pressure. sICH: symptomatic intracranial hemorrhage. STEMI/NSTEMI: ST-elevation/non ST-elevation myocardial infarction. TAVI: transcatheter aortic valve implantation. T2D: type 2 diabetes mellitus. TIA: transient ischaemic attack. VTE: venous thromboembolism.

**Supplemental Table 3. Prespecified or post-hoc subanalysis of clinical trials with positive results***

| **Trial acronym**/NCT** | **Trial/Population** | **Treatment** | **Primary endpoint** | **Results (HR; 95% CI; P value)** |
| --- | --- | --- | --- | --- |
| **1. Antihypertensives** | | | | |
| Intensive BP control versus standard BP control^14^ | Post-hoc, pooled participant-level analysis of 6 RCTs. 80220 assigned to intensive BP treatment (SBP target <120 mm Hg or <130 mm Hg) or standard treatment (SBP target <140 mm Hg, <150 mm Hg in older adults, or usual care). Female: 51.3%. FU: 3.2 years | Intensive BP control group (40,503) vs. standard BP control group (39,717) | Composite of MI, stroke, HF, and CV death. Primary harm outcomes: hypotension, syncope and renal-related events | Intensive BP control decreased the composite outcome vs. standard BP control (5.3% vs. 7.1%; 0.76, 0.72-0.81; P<0.0001). Intensive as compared with standard BP control resulted in an absolute risk reduction of 1.73% for CVD events (NNT 58), primarily driven by stroke reduction, and in an absolute risk increase of 1.82% in AEs (NNH 55), and showed a favourable benefit–harm profile, with a net benefit of 1.14, which persists when considering kidney-related AEs |
| Extended follow-up of the STEP trial^15^ NCT03015311 | After the original trial ended, all surviving patients, either in the standard group or the intensive treatment group previously, received intensive treatment in the extended period, referred to as the delayed intensive treatment group (DITG) or the sustained intensive treatment group (SITG). Female: 53.5%. FU: 6.11 years | Intensive treatment (SBP target of 110 mm Hg to <130 mmHg) vs. standard treatment (SBP target of 130 mm Hg to <150 mm Hg) | Composite of stroke, ACS, acute decompensated HF, coronary revascularization, atrial fibrillation, or CV death | The cumulative incidence for the primary outcome was 1.05% and 1.35% per year in the SITG and in the DITG (0.76; 0.62-0.93; P=0.009). There were no differences in safety between the 2 groups, but hypotension was more frequent in the SITG (3.82% vs. 2.81%; P=0.011). Compared with the standard BP treatment, intensive treatment initiating from randomization (0 month) yielded the greatest benefit (RR 0.83), with an attenuated CV benefit for later initiation: 12 months (0.88), 48 months (0.97) and 72 months (1.00) |
| **2. Antithrombotics** | | | | |
| **2.1. Anticoagulants** | | | | |
| COMBINE-AF^16^ Substudy | 5,913 who were frail, elderly patients (age ≥75 years) and VKA-experienced (FEV group) and 52,721 patients who did not meet all 3 of these criteria (control) group. FU: 27 months | Standard-dose (SD) DOAC or warfarin | Stroke or systemic embolic events, bleeding events, death, and a net clinical outcome combining these events | There was no heterogeneity in treatment effect with SD-DOAC vs warfarin between the two groups for the primary efficacy endpoints. Major bleeding was similar with SD-DOAC vs warfarin in the FEV group, but it was reduced with SD-DOAC in the control group (0.82; 0.76-0.89]; *P_int_*=0.007). Fatal and ICH were reduced with SD-DOAC in both subgroups to a similar degree (both *P_int_* >0.05), while GI bleeding with SD-DOAC increased in the FEV group compared with the control group (HR 1,83 vs. 1.23; *P_int_* = 0.006). The net clinical outcome was similar in the FEV group with SD-DOAC vs warfarin, but was significantly reduced with SD-DOAC in the control group (0.89; 0.85-0.93; *P_int_* = 0.028) |
| Oral Anticoagulation and Risk of Adverse Clinical Outcomes in Venous Thromboembolism^17^ | Population-based cohort study. 163593 patients with VTE who initiated an OAC therapy. Female: 56.7%. FU: until outcome occurrence, treatment discontinuation/switch, disenrollment or death | Apixaban (58.5%) vs. rivaroxaban (25.7%) vs. warfarin (15.8%) | Hospitalization for recurrent VTE and major bleeding | Compared with warfarin, patients taking apixaban (0.67; 0.61-0.75) and rivaroxaban (0.77; 0.69-0.87) had a lower risk of recurrent VTE. Apixaban showed a lower risk of major bleeding compared with warfarin (0.70; 0.64-0.76) and rivaroxaban (0.69; 0.63-0.75), but no difference in bleeding risk was observed between rivaroxaban and warfarin |
| **3. Beta-blockers** | | | | |
| Meta-analysis of β-blockers after MI with mildly reduced LVEF^18^  CRD420251023480 | Meta-analysis of 4 RCTs. 1885 patients with MI and mildly reduced LVEF (40-49%) and no history or signs of HF from the REBOOT, BETAMI-DANBLOCK and CAPITAL-trials. Median Female: 19%. FU: > 1 year | β-blocker therapy (initiated within 14 days post-MI) vs. non β-blocker therapy | Composite of ACD, new MI, or HF | Long-term oral β-blocker therapy reduced the primary endpoint as compared to the non β-blocker group (32.6 vs. 43.0 events per 1000 patient-years: HR 0.75; 0.58-0.97; P=0.031). There was no heterogeneity between the trials (trial-by-treatment P_int_=0.95) or countries of enrolment (P_int_=0.98) |
| **4. Cardiomyopathies: Hypertrophic cardiomyopathy (HCM), Transthyretin amyloid cardiomyopathy (ATTR-CM)** | | | | |
| Post-hoc analysis of the ATTRibute-CM trial^19^  NCT03860935 | Phase 3, DB, R, controlled trial. 632 with symptomatic ATTR-CM,  NYHA functional class I_III, and either a wild-type or a variant TTR genotype. Female: 9.2%. FU: 30 months | Acoramidis (800 mg bid) or placebo for 30 months | Hierarchical combination of ACD, CV-related  hospitalization (CVH), NT- proBNP level, and 6-MWD. | Compared with placebo, acoramidis reduced the primary endpoint (35.9% vs. 50.5%; 0.64; 0.50-0.83; P=0.0008) and the first CVH (26.7% vs. 42.6%; 0.60; 0.45-0.80; P=0.0005), with Kaplan-Meier curves separating at month 3. The annualized frequency of CVH was reduced with acoramidis vs. placebo (0.22 vs. 0.45; relative risk ratio: 50%; 0.36-0.70; P<0.0001) and the efficacy of acoramidis on the composite of ACM or first CVH was consistent across subgroups. No safety signals of clinical concern were identified. |
| ATTRibute-CM trial. Open-label extension^20^  NCT04988386 | Phase 3, R, controlled trial. 389 with ATTR-CM who received acoramidis through month 30 continued to receive it; those who received placebo through month 30 were switched to acoramidis for 12 months. Tafamidis was discontinued. Female: 8%. FU: 42 months | Acoramidis (800 mg equivalent to acoramidis 712 mg bid) or placebo | Clinical efficacy outcomes | At month 42, continuous acoramidis reduced ACM or first CV related hospitalization (42.5% vs. 64.4%; HR 0.57; 0.46-0.72; P<0.001), ACM alone (0.64; 0.47-0.88) and first CV hospitalization alone (0.53; 0.41-0.69). Treatment effects for NT-proBNP, 6-MWD and QoL assessed by KCCQ-OSS also favoured continuous acoramidis. No new clinically important AEs were identified |
| Post hoc analysis of the ATTRibute-CM and its open-label extension^21^  NCT03860935 | 611 participants from the ATTRibute-CM trial were included in this analysis. Female: 9%. FU: 42 months | Acoramidis (800 mg equivalent to acoramidis 712 mg bid) or placebo | Cumulative incidence of CV outcomes from ATTRibute-CM and its open-label extension through month 42 | Acoramidis compared with placebo reduced the burden of CV outcomes (0.51; 0.43-0.62; *P*<0.0001) and the risk of recurrent CV hospitalization (0.50; 0.35-0.69; *P<*0.0001) over 30 months vs. placebo. At month 42, CVM was reduced with continuous acoramidis vs placebo-to-acoramidis (0.55; 0.39-0.79; *P=*0.0011) |
| Substudy of the VANISH trial^22^  [NCT01912534](https://pubmed.ncbi.nlm.nih.gov/34556856/) | Phase 2, DB, PC trial. 137 with early stage sarcomeric HCM (aged 8 and 45 years) with no or minimal symptoms underwent CMR imaging at baseline and year 2. Female: 37.2%. FU: 2 years | Valsartan (80 mg/day for children weighing <35 kg, 160 mg/day for those weighing ≥35 kg, or 320 mg/day in adults) vs. placebo | Mean change in CMR parameters between baseline and year 2, including indexed extracellular (iECV) and intracellular volume (iICV), and late gadolinium enhancement (LGE) | Valsartan increased LV end-diastolic volume index (mean difference 3.3 mL/m2; 0.4-6.2; P=0.03) but did not modify LV mass index or LVEF. It appeared to reduce the decline in right ventricular volumes and significantly reduced iICV progression (mean difference -5.0 mL/m^2^; P=0.03), but did not impact iECV or LGE progression |
| **5. Incretin therapy** | | | | |
| Prespecified analyses of the SOUL trial^23^  NCT03914326 | Patients receiving SGLT2i at baseline (2596) and subsequently during the trial (4718). Females: 26.5%. FU: 47.5 months | Semaglutide (3 mg up to 14 mg ) or placebo od | Time to first major adverse CV event, defined as CV death, nonfatal MI, or nonfatal stroke. | Semaglutide reduces MACE event outcomes consistently in participants with (0.89; 0.71-1.11) and without concomitant SGLT2i use (0.84; 0.74-0.95), and the combination appears to be safe |
| SUMMIT prespecified analysis^24^  NCT04847557 | Phase 3, R, DB, PC trial. 731 with HFpEF, BMI≥30 kg/m^2,^ who were enriched for participants with CKD.. FU: 104 weeks | SC tirzepatide (2.5 up to 15 mg/week) or placebo | Time to first CV death or worsening HF event; and change in the KCCQ-CSS at 52 weeks | CV death or worsening HF events occurred less frequently in the tirzepatide group (0.62; 0.41-0.95; P=0.026), primarily related to fewer worsening HF events. The effect on the primary endpoint iwas similar in patients with/without T2D (P_int=_0.95). Weight loss was less pronounced in patients with than without T2D (10.4% vs 12.9%; P_int=_0.04), but both groups showed similar decreases in visceral adiposity |
| SUMMIT^25^  NCT04847557 | Phase 3, R, DB, PC trial. 731 with HFpEF, BMI≥30 kg/m^2,^ who were enriched for participants with CKD.. FU: 52 weeks |  | Influence of CKD on the clinical responses to tirzepatide in patients with obesity-related HFpEF and complexity of tirzepatide-related changes in renal function after 52 weeks | CKD did not inﬂuence the benefitst of tirzepatide on major adverse HF events, KCCQ-CSS, exercise tolerance, functional capacity, and QoL, but the absolute risk reduction was numerically greater in patients with CKD. At 52 weeks, tirzepatide improved the eGFR assessed by cystatin C in all patients, but only in patients with CKD when assessed by eGFR-creatinine. Tirzepatide produced a similar decrease in visceral adiposity and LV mass in patients with/without T2D. |
| SUMMIT Trial^26^  NCT04847557 | Phase 3, R, DB, PA trial. 731 with class II-IV HFpEF and obesity (BMI ≥30 kg/m^2^). Female: 53.8%. FU: 52 weeks | Tirzepatide (2.5 mg SC titrated to 10 or 15 mg QW) | Combined risk of CV death or worsening HF and improved KCCQ-CCS | Tirzepatide reduced the risk of the composite of CV death or worsening HF events (0.62; 0.41–0.95; *P*=0.026) and ACD or worsening HF events (0.67; 0.46–0.99; *P*=0.045). The benefit was driven by an effect on worsening HF events requiring hospitalization or the use of IV medications in an urgent care setting (0.41; 0.22–0.75; *P*=0.004). Tirzepatide also improved NYHA class and increased the KCCQ-CSS, 6-MWD, and EQ-5D-5L score (all P<0.001) |
| SUMMIT^27^ | 731 with class II-IV HFpEF and BMI ≥30 kg/m^2^ |  | Time to CV or worsening HF and change in KCCQ-CSS at 52 weeks. | Tirzepatide reduced risk of HF events or CV death in patients with obesity-related HFpEF regardless of baseline BMI or fat distribution. However, greater weight loss was associated with larger improvements in 6-MWD, KCCQ-CSS, CRP, and SBP, and a greater decrease in waist circumference with larger increases in 6-MWD and KCCQ-CSS |
| SURMOUNT-1^28^  [NCT04184622](http://clinicaltrials.gov/show/NCT04184622) | Phase 3, DB, R, controlled trial. 2539 with obesity and prediabetes. Female: 64%. FU: 176 weeks, followed by a 17-week off-treatment period. | Tirzepatide at a once-weekly dose of 5 mg, 10 mg, or 15 mg  or placebo | Percent of change in BW and percentage of participants with ≥5% weight reduction | At 176 weeks, the mean percent change in BW was −12.3%, -18.7% and −19.7% in patients treated with 5, 10 and 15-mg of tirzepatide (−1.3% with placebo; all P<0.001 vs. placebo) and fewer patients received a diagnosis of T2D in the tirzepatide groups than in the placebo group (1.3% vs. 13.3%; P<0.001). After 17 weeks off treatment, 2.4% who received tirzepatide and 13.7% of those who received placebo had T2D (0.12; P<0.001). |
| **6. Lipid-lowering drugs** | | | | |
| Analysis of the FOURIER trial^29^ | Phase 3, R, DB, PC trial. 27564 with stable ASCVD, 889 with and 26675 without any autoimmune or inflammatory disease (AIID). Female: 30%. FU: 2.2 years | SC evolocumab (140 mg Q2W or 420 mg monthly) or placebo. | Composite of CV death, MI, stroke, hospitalization for unstable angina, or coronary revascularization | Compared with placebo, evolocumab reduced the rate of the primary end point in patients without (0.86; 0.80– 0.93) and with an AIID (0.58; 0.38–0.89; P_int_=0.066) and the secondary end point of CV death, MI, or stroke in patients without (0.81; 0.74-0.89) and with AIID (0.42; 0.24-0.74; P_int_=0.022). The effect of evolocumab on LDL-C levels was consistent irrespective of AIID status |
| Subanalysis of the STOP-CA trial^30^  NCT02943590 | DB, R trial. 300 with recent diagnosis of lymphoma. Female: 47%. FU: 12 months | Atorvastatin (40 mg od) or placebo | Cardiac magnetic resonance imaging–derived aortic arch pulse wave velocity (PWV) and ascending aortic distensibility (AAD) | At 12 months, PWV values remained similar in the atorvastatin group (6.5 vs. 6.5 m/s) but increased in the placebo group (5.7 vs 6.8 m/s). A 1 SD or more increase (0.8 m/s) in PWV was observed among 5% patients with atorvastatin (50% of patients with placebo; P<0.001) and a 1 SD or more decrease (1.8 × 10^−3^ mm Hg^−1^) in AAD in 7% and 18% of patients, respectively. A higher 12-month interval increase in PWV was associated with a larger subsequent decrease in LVEF |
| **7. Renin-angiotensin-aldosterone inhibitors** | | | | |
| FINE-HEART^31^  (CRD42024570467) | Prospectively planned pooled analysis. 14,581 with CKD and T2D (FIDELIO-DKD and FIGARO-DKD trials) and HFmrEF or HFpEF (FINEARTS-HF trial). Among participants without AF/AFL at baseline, 631 experienced new-onset AF/AFL during follow-up (2.9 years). Females: 33% | Finerenone vs. placebo | New-onset AF/AFL was prospectively adjudicated in all 3 trials by a blinded independent clinical endpoint committee | Finerenone reduced the incidence of new-onset AF/AFL across the CKM spectrum (3.9% vs. 4.7%; HR 0.83; 0.71-0.97; P=0.019), irrespective of number of CKM conditions. Patients who experienced new-onset AF/AFL had a higher subsequent risk for HF hospitalization or CV death, MACE, kidney composite outcome and all-cause death (all P<0.001) than those who remained free of AF/AFL during follow-up |

***** In alphabetic order ** Acronyms of the trials are summarized in Supplemental Table 7

Abbreviations. ACD: all-cause death. AF: atrial fibrillation. AFL: atrial flutter. ASCVD: atherosclerotic cardiovascular disease. BMI: body mass index. BP: blood pressure. CI: confidence interval. CKD: chronic kidney disease. CKM: cardiovascular-kidney-metabolic. CMR: cardiac magnetic resonance. CRP: C-reactive protein. CV: cardiovascular. DB; double blind. DBP: diastolic blood pressure. DOAC: direct oral anticoagulant. FU: follow-up. eGFR: estimated glomerular filtration rate. HDL-C: high-density lipoprotein cholesterol. HR: hazard ratio. HF: heart failure. HFrEF/HFmrEF/HFpEF: heart failure with reduced, mildly reduced or preserved ejection fraction. HR: heart rate. ICH: intracranial hemorrhage. IV: intravenous. KCCQ-CSS: Kansas City Cardiomyopathy Questionnaire-Clinical Summary Score. LDL-C: low-density lipoprotein cholesterol. LGE: late gadolinium enhancement. LV: left ventricular. LVEF: left ventricular ejection fraction. MACE: major cardiovascular events. MI: myocardial infarction. 6-MWD: 6-minute walk distance. NCT: ClinicalTrials.gov identifier. NNH/NNT: number needed to harm/treat. NT-proBNP: N-terminal pro-B-type natriuretic peptide. NYHA: New York Heart Association. OAC: oral anticoagulant. od: once daily. OL: open label. PA: parallel assignment. PC: placebo-controlled. PCI: percutaneous coronary intervention. QoL: Quality of Life. QW: once weekly. Q2W: every 2 weeks. R: randomized. RCTs: randomized clinical trials. SBP: systolic blood pressure. SC: subcutaneous. SGLT2i: sodium-glucose cotransporter 2 inhibitors. T2D: type 2 diabetes mellitus. VTE: venous thromboembolism.

# **Supplemental Table 4. Clinical trials with neutral or negative results***

| **Trial acronym**/NCT** | **Trial/Population** | **Treatment** | **Primary endpoint** | **Results (HR, 95% CI)** |
| --- | --- | --- | --- | --- |
| **1. Antithrombotic drugs** | | | | |
| AQUATIC^32^  NCT04217447 | Phase 3, P, DB, R, OC trial. 872 with CAD who underwent stent implantation >6 months before enrollment who were at high risk for atherothrombotic events. Female: 85.3%. FU 2.2 years | Aspirin (100 mg 0d) or placebo. All patients continued their current OAC therapy | Composite of CV death, MI, stroke, systemic embolism, coronary revascularization, or acute limb ischemia | The addition of aspirin increased the primary endpoint (16.9% vs. 12.1%; 1.53; 1.07-2.18; P=0.02), all-cause death (13.4% vs. 8.4%; 1.72; 1.14-2.58; P=0.01), major bleeding (10.2% vs. 3.4%; HR 3.35; 1.87-6.00; P<0.001) and serious AEs (28.6% vs. 17.3%; 1.85; 1.39-2.46; P<0.001). These results do not support the addition of aspirin to OAC therapy in these patients. |
| DOAC-CVT^33^  [NCT04660747](https://clinicaltrials.gov/show/NCT04660747) | P, observational cohort study. 619 with radiologically confirmed CVT starting oral ACT, as per local practice, within 30 days after diagnosis. Female: 63%. FU: 6 months | DOACs vs VKAs | Composite of symptomatic VTE and major bleeding events (ISTH criteria) at 6 months | The rate of recurrent venous thrombosis and major bleeding did not differ between patients treated with DOACs vs. VKAs (OR 0.99; 0.37-3.38). Thus, DOACs are a reasonable alternative to VKAs for CVT |
| HOST-BR^34^  NCT05631769 | Phase 4, R, OL trial. 4897 who received PCI with a drug-eluting stent. One-third had HBR and two-thirds LBR. Female: 21.5%. FU: 1 year | DAPT: 1 month vs. 3 months in the HBR group, and 3 vs. 12 months in the LBR group. Use of specific P2Y12 inhibitor was at the physician’s discretion | Net adverse clinical events (all-cause death, MI, stent thrombosis, stroke, or major bleeding), MACCE (cardiovascular death, MI, definite or probable stent thrombosis, or ischaemic stroke), and any actionable non-surgical bleeding at 1 year after randomisation | In the HBR group, 1-month DAPT did not reach non-inferiority compared to 3-month DAPT for net adverse clinical events (18.4% vs. 18.4%; HR 1.33; 1.04-1.73; P=0.82 for non-inferiority); bleeding occurred in 13.8% and 15.8% patients in the 1- and 3-month groups, respectively. In the non-HBR stratum, 3-month was non-inferior to 12-month DAPT regarding net adverse clinical events (2.9% vs. 4.4%; 0.65; 0.45-0.94; P<0.0001 for non-inferiority) and major adverse cardiac or cerebral events (2.2% vs. 2.3%; P=0.0082 for non-inferiority), and superior for bleeding (7.4% vs. 11.7%; 0.63; 0.50-0.79; P<0·0001) |
| NEOMINDSET^35^  NCT04360720 | Phase 3, OL, R trial with blinded outcome adjudication. 3,410, with ACS who had undergone successful PCI with drug-eluting stents. Female: 29.3%. FU: 12 months | Potent P2Y12 inhibitor monotherapy (ticagrelor 5-10 mg od or prasugrel 90 mg bid) vs. DAPT (aspirin, 80-100 mg od, plus a potent 2Y12 inhibitor) | Composite of all-cause death, MI, stroke, or urgent target-vessel revascularization and major or clinically relevant nonmajor bleeding | At 12 months, potent P2Y12 inhibitor monotherapy was not noninferior to DAPT (7% vs. 5.5%; HR 1.28; 0.98-1.68) and the absolute difference in risk (1.47 percentage points) did not meet the prespecified criterion for noninferiority (P=0.11). Hemorrhagic events were less frequent in the monotherapy group than in the DAPT group (2.0% vs. 4.9%) |
| OCEAN Investigators^36^  NCT02168829 | Phase 4, OL, R, blinded-outcome-assessment trial. 1284 who underwent successful catheter ablation for AF at least 1 year earlier, with CHA2DS2-VASc score ≥1 (≥2 for women or for patients in whom vascular disease was a risk factor). Females: 28.6%. FU: 3 years | Aspirin (70 to 120 mg od, depending on availability in the local jurisdiction) or rivaroxaban (15 mg od) | Composite of stroke, systemic embolism, or new covert embolic stroke (defined by ≥1 new infarct measuring ≥15 mm on MRI) at 3 years | Rivaroxaban did not reduce the primary endpoint as compared with aspirin (0.31 vs. 0.66 events per 100 patient-years, respectively; absolute risk difference at 3 years, −0.6%; −1.8 to 0.5; P=0.28) or in cerebral infarcts <15 mm. Major bleeding, minor bleeding and clinically relevant nonmajor bleeding occurred in more patients treated with rivaroxaban than with aspirin (1.6% vs. 0.6%, 11.5% vs. 3.2%, and 5.5% vs 1.6%, respectively) |
| START^37^  NCT03021928 | Phase 2, pragmatic, response-adaptive randomized trial. 200 with mild-to-moderate ischemic stroke (minimum lesion diameter of 1.5 cm) with AF and receiving a DOAC within 2 weeks from stroke onset. Female: 50%. FU: 30 days | DOAC treatment started day 3-4, 6, 10 or 14 after stroke onset | Ischemic (stroke or systemic embolism) or haemorrhagic (symptomatic intracranial or major systemic hemorrhage) event observed within 30 days from the index stroke time of onset | A clearly superior day to initiate use of a DOAC was not identified, but the evidence suggested that initiating a direct oral anticoagulant earlier is better than later within the first 2 weeks after stroke due to AF. |
| TACSI^38^  NCT03560310 | Phase 4, OL, registry-based trial. 2201 patients who underwent CABG for an ACS. Female: 14.4%. FU: 1 year | DAPT with ticagrelor (90 mg bid) plus aspirin (75-100 mg daily) or aspi­rin alone (75 to 160 mg daily according to local practice) | MACE, composite of death, MI, stroke, or repeat revascularization, evaluated at 1 year | DAPT did not reduce the incidence of the primary end point than treatment with aspirin alone (4.8% vs.4.6%; 1.06; 0.72-1.56; P=0.77). Net adverse clinical events occurred in 9.1% of patients in the DAPT group and 6.4% in the aspirin-alone group (1.45; 1.07-1.97). Major bleeding occurred in more patients in the DAPT group than in the aspirin group (4.9% vs. 2.0%; 2.50; 1.52-4.11) |
| TADCLOT^39^  NCT06318481 | Phase 3, DB, R, superiority trial. 2.201 patients with STEMI within 24 hours of primary PCI. FU: 1 months | Ticagrelor (180 mg loading dose; then 90 mg bid) or clopidogrel (600 mg loading dose; then dose 75 mg bid) for 1 month | MACE (death, MI, stent thrombosis, stroke, or target lesion revascularization) at 1 month, analysed by intention to treat. | Ticagrelor was not superior to clopidogrel in reducing MACE (2.2% vs 2.9%; HR: 0.75; 0.44-1.27; P=0.28; absolute risk difference: -0.7%; -2.05 to 0.60), CVD or definite stent thrombosis occurred after primary PCI. MACE was significantly lower with ticagrelor compared with clopidogrel at both 7 (0.3% vs. 1.8%; *P*=0.002) and 14 days (1.1% vs. 2.4%; *P=*0.02), but not at 30 days (2.2% vs. 2.9%; 0.75; 0.44-1.27; *P*=0.28). Major bleeding (BARC type 3 or 5) was similar between the groups |
| TAILORED-CHIP^40^  NCT03465644 | Phase 4, OL, R, PA trial. 2018 with high-risk anatomical or clinical characteristics undergoing complex PCI. Female: 17%. FU: 12 months | Early (<6 months post-PCI) escalation with ticagrelor (120 mg loading, then 60 mg bid) plus aspirin vs. late (>6-month post-PCI) de-escalation with clopidogrel alone as compared with DAPT (clopidogrel plus aspirin) for 12 months | Net adverse clinical events, a composite of ACD, MI, stroke, stent thrombosis, unplanned urgent revascularization, and clinically relevant bleeding (BARC Type 2, 3, or 5) at 12 months | At 12 months, there were no differences between the tailored-therapy and DAPT groups in the primary outcome (10.5% vs. 8.8%; HR 1.19; 0.90-1.58; P=0.21) or in the incidence of major ischaemic events (3.9% vs. 5.0%; 0.78; 0.52-1.19; P=0.25). The incidence of clinically relevant bleeding was higher in the tailored-therapy group compared with the DAPT group (7.2% vs. 4.8%; P=0.002), but the incidence of major bleeding was similar in both groups |
| **2. Colchicine** | | | | |
| CLEAR^41^  NCT03048825 | Phase 4, 2x2 factorial, R, controlled trial. 7062 with previous MI Females: 205%. FU: 3 years | Colchicine (0.5 mg/day) vs. placebo | Composite of CV death, recurrent MI, stroke, or unplanned ischemia-driven coronary revascularization, evaluated in a time-to-event analysis | Colchicine did not reduce the incidence of the primary outcome (9.1% vs.9.3%; HR 0.99; 0.85-1.16; P=0.93). Diarrhea was more frequent in the colchine than in the placebo group (10.2% vs. 6.6%; P<0.001), but not serious infections between groups |
| COCOMO trial^42^  ACTRN12618000809235 | Investigator-initiated, R, DB, PC trial. 64 with non-ST elevation MI and a non-culprit coronary artery that contained at least one lipid-rich plaque causing ≥20% stenosis. Female: 9.4%. FU: 17.8 months | Colchicine 0.5 mg/day or placebo | Change in minimum fibrous cap thickness (FCT) in non-culprit segments from baseline to final visit | Colchicine had no effect on the minimal FCT or the maximum lipid arc throughout the imaged non-culprit segment post-MI (both P=0.18). However, cap rupture was less frequent in the colchicine group (3.6% vs 27.6% with placebo; P=0.03). In 43 patients followed for 16 months, the minimum FCT increased to a greater extent in the colchicine group vs. placebo (+64.7 vs +38.7; P=0.005). |
| **3. Diuretics** | | | | |
| CLEAR investigators^43^  NCT03048825 | Phase 3, 2x2 factorial design, R, controlled trial. 7062 patients with large NSTEMI who had undergone PCI. Female: 20%. FU: 3 years. | Spironolactone (25 mg od) or placebo | Composite of CV death or new or worsening HF; and a composite of the first occurrence of MI, stroke, new or worsening HF, or cardiovascular death | Over a median FU of 23 years, as compared with placebo, spironolactone did not reduce first (HR 0.91; 0.69-1.21; P=0.51) and second primary outcome (0.96; 0.81-1.13; P=0.60) |
| **4. Glucose-lowering drugs** | | | | |
| PERMET^44^  NCT03054519 | Phase 4, R, DB trial. 202 with peripheral artery disease (ankle-brachial index ≤0.90 or toe-brachial index ≤0.70) without diabetes. Female: 28%. FU: 6 months | Metformin (2000 mg daily taken as 2 pills bid) or placebo 2 pills bid) | 6-month change in 6-min walk distance (minimum clinically important difference, 8 to 20 m) | Metformin did not improve 6-minute walk distance compared with placebo (metformin, within-group change: −5.4 m; placebo: within-group change: −5.3 m; mean between-group difference 1.1 m; −16.3 to 18.6 m; P=0.9) |
| **5. Heart failure drugs** | | | | |
| Beta-Blocker Trialists’ Collaboration Study Group^45^  CRD420251119176 | Meta-analysis of 5 open-label trials. 17,801 patients with a preserved LVEF patients with recent MI, no other indications for β-blocker therapy, and an LVEF of at ≥50%. Female: 20.6%. FU: 3.6 years | β-blocker therapy vs. no β-blocker therapy | Composite of ACD, MI, or HF | β-blocker therapy did not reduce the incidence of the primary endpoint (HR 0.97; 0.87-1.07; P=0.54) or any of its components, in patients with an LVEF ≥50% after MI without other indications for β-blockers |
| FAIR-HF2 DZHK05^46^  NCT03036462 | Phase 4, P, R trial. 1105 with HFrEF and iron deficiency (serum ferritin level <100 ng/mL; or if transferrin saturation was <20%, had serum ferritin levels between 100-299 ng/mL). Female: 33%. FU: 16.6 months | Ferric carboxymaltose (IV dose of up to 2000 mg, followed by 500 mg every 4 months) vs a saline placebo | (1) Time to CV or first HFH, (2) total HFH, and (3) time to CV death or first HFH in patients with a transferrin saturation <20%. | Ferric carboxymaltose did not reduce the time to first HFH or CV in the overall cohort (HR 0.79; 0.63-0.99; P=0.04) or in patients with a transferrin saturation <20% (0.79; 0.61-1.02; P=0.07), and the total number of HFH vs placebo (RR 0.80; 0.60-1.06; P=0.12). |
| REBOOT-CNIC^47^  NCT03596385 | OL, R trial. 8505 with AMI (STEMI/NSTEMI) that received invasive care and a LVEF >40%. Females: 19.2%. FU: 3.7 years | β-blocker (bisoprolol 86%; dose at physician discretion) vs. no β-blocker therapy | Composite of death from any cause, reinfarction, or HF hospitalization | β-blocker therapy did not modify the primary endpoint (22.5 vs. 21.7 events per 1000 patient-years; HR 1.04; 0.89-1.22; P=0.63), death from any cause (1.06; 0.85-1.33); reinfarction (1.01; 0.80-1.27); and HFH (0.89; 0.58-1.38). No differences in safety outcomes between groups were noted. |
| VICTOR^48^  NCT05093933 | Phase 3, DB, PC trial. 10921 with HFrEF without HFH within 6 months or outpatient IV diuretic use within 3 months before randomization. Female: 23.6%. FU: 18.5 months (19.7 months for mortality) | Oral vericiguat (2.5 mg up to 10 mg od) vs. placebo | Cardiovascular death or HFH | At 18.5 months, vericiguat did not reduce the risk of cardiovascular death or HFH vs. placebo (18.0% vs. 19.1%; HR 0.93; 0.83-1.04; P=0·22). However, fewer cardiovascular (9.6% vs. 11.3%) and ACD (secondary endpoints) occurred in the vericiguat group than in the placebo group (12.3% vs. 14.4%) |
| **6. Lipid-lowering drugs** | | | | |
| CAVIAR^49^  NCT03537742 | Phase 2, investigator-initiated, P, DB, R trial. 114 within 6 months of heart transplantation recipients. Female: 20%. FU: 1 year | Patients receiving rosuvastatin (10 or 20 mg od) were randomized to alirocumab (initial dose: 150 mg SC Q2W) or placebo | Change in coronary artery plaque volume from baseline to one year post HT based on serial IVUS. | At 12 months, LDL-C levels decreased in the alirocumab arm (from 72.7 ±31.7 to 31.5 ±20.7 mg/dL), but not in the placebo group. However, plaque volume increased numerically in both groups and the change in plaque volume did not differ between groups (P=0.86). Fractional flow reserve, coronary flow reserve and the index of microcirculatory resistance did not change significantly with the addition of alirocumab |
| NEWTON-CABG CardioLink-5^50^  [NCT03900026](https://clinicaltrials.gov/show/NCT03900026) | Phase 4, DB, R, PC trial. 554 who underwent CABG with at least two SVG and treated with statin therapy of moderate or high intensity. Female: 15%. FU: 24 months | Evolocumab 140 mg or placebo SC every 2 weeks | 4-month vein graft disease rate (proportion of SVGs with ≥50% occlusion on coronary CT angiography or clinically indicated invasive angiography) | Evolocumab did not reduce SVG disease rate at 24 months following the index surgery (21.7% vs. 19.7%; P=0.44) despite substantial placebo-adjusted reduction in LDL-C at 24 months (-52·4% vs -4·0%). |
| **7. Mavacamten** | | | | |
| ODYSSEY-HCM^51^ NCT05582395 | Phase 3, DB, PC trial. 580 with symptomatic nonobstructive HCM. Females: 46%. FU: 48 weeks. | Mavacamten (starting at 5 mg od adjusted up to a maximum of 15 mg depending on the LVEF or placebo | Change from baseline to week 48 in peak oxygen uptake and symptoms (assessed by the KCCQ-CSS) | At 48 weeks, mavacamten did not improve peak oxygen uptake (0.52 vs 0.05 mL/kg/min) or decreased symptoms (KCCQ-CSS: 13.1 vs. 10.4 points; P=0.06) as compared with placebo. Reductions in ejection fraction (LVEF <50%: 21.5% vs 1.7%; LVEF ≤30%: 2.4% vs. 0%), AF (6.6% vs. 4.2%) and interrup­tions in the trial regimen (25.7% vs. 7.6%) were more common with mavacamten than with placebo |
| **8. Sodium-glucose cotransporter 2 (SGLT2) inhibitors** | | | | |
| DAPA ACT HF-TIMI 68^52^  NCT04363697 | Phase 4, R, DB, PC trial. 2401 who have been stabilized during hospitalization for acute HF. Female: 33.9%. FU: 2 months. | Dapagliflozin (10 m/day) at least 24 h and no later than 14 days after hospital admission vs. placebo | Composite of time to CV death or worsening HF over the first 2 months | In-hospital initiation of dapagliflozin did not reduce the primary endpoint (10.9% vs. 12.7%; HR 0.86; 0.68-1.08; P=0.20), CV death (2.5% vs. 3.1%; 0.78; 0.48-1.27), worsening HF (9.4% vs. 10.3%; 0.91; 0.71-1.18) and ACD (3.0% vs. 4.5%; 0.66; 0.43-1.00). Symptomatic hypotension and rates of worsening kidney function were higher with dapagliflozin vs. placebo (3.6% vs. 2.2% and 5.9% vs. 4.7%, respectively) |

***** In alphabetic order ** Acronyms of the trials are summarized in Supplemental Table 7.

Abbreviations. ACD: all-cause death. AE: adverse event. AF: atrial fibrillation. AMI: acute myocardial infarction. BARC: Bleeding Academic Research Consortium. bid: twice daily. CAD: coronary artery disease. CAGB: coronary artery bypass graft. CI: confidence interval. CV: cardiovascular. CVT: cerebral venous thrombosis. DAPT: dual antiplatelet therapy. DB; double blind. DOAC: direct oral anticoagulant. FU: follow-up. HR: hazard ratio. HF: heart failure. HFH: heart failure hospitalization. HFrEF: heart failure with reduced ejection fraction. ISTH: International Society on Thrombosis and Haemostasis criteria. IV: intravenous. IVUS: intravascular ultrasound. KCCQ-CSS: Kansas City Cardiomyopathy Questionnaire-Clinical Summary Score. LDL-C: low-density lipoprotein cholesterol. LVEF: left ventricular ejection fraction. MACE: major cardiovascular events. MI: myocardial infarction. MRI: magnetic resonance imaging. NCT: ClinicalTrials.gov identifier. od: once daily. OL: open label. PA: parallel assignment. PC: placebo-controlled. PCI: percutaneous coronary intervention. Q2W: every 2 weeks. R: randomized. SC: subcutaneous. STEMI/NSTEMI: ST-elevation/non ST-elevation myocardial infarction. SVT: saphenous vein graft. VKAs: vitamin K antagonists. VTE: venous thromboembolism.

**Supplemental Table 5. Recent phase 2 and 3 trials with some cardiovascular drugs under clinical development**

| **Trial/Acronym/NCT** | **Trial/Population** | **Treatment** | **Primary endpoint** | **Results** |
| --- | --- | --- | --- | --- |
| **1. Antiobesity drugs** | | | | |
| ACHIEVE-1^53^  NCT05971940 | Phase 3, DB, R, PC trial. 559 with T2D, HbA1c ≥7.0% (mean 8%), but no more than 9.5%, and a BMI ≥23.0. Female: 48%. FU: 40 weeks | Orforglipron (3, 12 or 36 mg) or placebo od for 40 weeks | Change from baseline to week 40 in the HbA1c level | At week 40, orforglipron reduced mean Hb1Ac -1.24%, -1.47% and -1.48%, respectively (-0.41 with placebo; all P<0.001), and target values <7.0% were reached in 68%-73% and values <5.7% in up to 24% of participants. Orforglipron also reduced BW (-4.5% to -7.6% vs. -1.7% with placebo; P<0.001) and mean fasting serum glucose (-31 mg/dL vs. −11 mg/dL with placebo; P<0.001). Discontinuation due to gastrointestinal AEs occurred in 2.2%-5.7% of participants in the orforglipron groups (0% with placebo). Ortoglipron increased pulse rate (2.2.-4.8 bpm) |
| ATTAIN-1^54^  NCT05869903 | Phase 3, R, DB trial. 3127 without diabetes and a BMI ≥30 or between 27 and 30with at least one obesity-related complication. Female: 64%. FU: 72 weeks | Orforglipron (6, 12, or 36 mg) or placebo | Percent change in BW from baseline to week 72, as assessed according to the treatment-regimen estimand in the intention-to-treat population | At week 72, the mean change in BW was -7.5%, -8.4% and with ortoglipron, and -2.1% with placebo (all P<0.001 vs. placebo). Among the patients in the orforglipron 36-mg group, 54.6% had a reduction ≥10%, 36.0% a reduction ≥15% and 18.4% a reduction ≥20% vs. 12.9%, 5.9%, and 2.8%, respectively, in the placebo group. Waist circumference, SBP, TG, and non-HDL cholesterol levels significantly improved with orforglipron treatment as compared with placebo. AEs resulting in treatment discontinuation appeared in 5.3-10.3% of the patients in the orforglipron groups and in 2.7% of those in the placebo group. |
| GLORY-1^55^  NCT05607680 | Phase 3, R, DB trial. 610 with a BMI ≥28 or a BMI of 24 to <28 plus at least one weight-related coexisting condition, Female: 51%. FU: 32 weeks | SC mazdutide (4 or 6 mg) or placebo once weekly | Percentage change in BW from baseline and a weight reduction ≥5% at week 32, as assessed in a treatment-policy estimand analysis | At week 32, the mean percentage change in BW from baseline was –10.09% and –12.55% with 4 and 6 mg of mazdutide and 0.45% in the placebo group; 73.9%, 82.0%, and 10.5% of the participants, respectively, had a BW ≥5% (all P<0.001 vs. placebo). At week 48, the mean percentage change in BW from baseline was –11.00%, –14.01% and 0.30% and 35.7%, 49.5%, and 2.0% of the participants, respectively, had a weight reduction ≥15% (all P<0.001 vs. placebo) |
| Maridebart cafraglutide  NCT05669599^56^ | Phase 2, DB, R, PC, dose-ranging trial that included 11 groups as two cohorts. 592 with obesity (Obesity cohort: mean BMI 37.9; female: 63%) and with obesity and T2D (obesity–diabetes cohort: mean BMI 36.5; female: 63%. FU: 52 weeks. | Obesity: maridebart cafraglutide SC (140, 280, or 420 mg Q4W or 420 mg Q8W without dose escalation; 420 mg Q4W with 4-week dose escalation; 420 mg Q4W with 12-week dose escalation; or placebo. Obesity–diabetes cohort: 140, 280, or 420 mg every 4 weeks without dose escalation or placebo | Percent change in BW from baseline to week 52 | Obesity cohort: mean percent change in BW from baseline to week 52 based on the efficacy estimand ranged from −12.3% to −16.2% (−2.5% with placebo); in the obesity–diabetes cohort ranged from −8.4% to −12.3% (−1.7% with placebo). Reductions in fat mass ranged from −26.2% to −36.8% in patients with obesity and −17% to −34% in patients with obesity and T2D (−9% and -4% with placebo, respectively. Maridebart cafraglutide lowered HbA1c decreased 1,6-2,2 percentage points (0.1 with placebo). Drug discontinuation due to GI AEs occurred in 12-27% of no-escalation groups and 8% of dose-escalation groups. |
| REDEFINE 1^57^  NCT05567796 | Phase 3a, DB, PC and active-controlled trial. 2108 without diabetes who had a BMI ≥30 or a BMI ≥27 with at least one obesity-related complication. Females. 67%. FU: 68 weeks | Once-weekly SC semaglutide (2.4 mg) cagrilintide (2.4 mg, semaglutide alone, cagrilintide alone, or placebo SC | Relative change in BW and a reduction of ≥5% in BW from baseline to week 68 as compared with placebo. | The estimated mean percent change in BW from baseline was –20.4% with cagrilintide–semaglutide and -3% with placebo (estimated difference, –17.3%; -18.1 to -16.6; P<0.001). Patients on cagrilintide–semaglutide were more likely than those on placebo to reach weight-loss ≥ 5%, ≥20% or ≥25% and ≥30% (all P<0.001). Gastrointestinal AEs (nausea, vomiting, diarrhea, constipation, abdominal pain) were re frequent in the cagrilintide–semaglutide (79.6%) than in the placebo group (39.9%) |
| REDEFINE 2^58^  NCT05394519 | Phase 3a, R, PC trial. 1206 with a BMI ≥27, HbA1c of 7-10% and T2D. Females. 47%. FU: 68 weeks | Once-weekly SC CagriSema (at a dose of 2.4 mg each) or placebo | Percent change in BW and the percentage of patients with a weight reduction of at least 5% | The estimated mean change in BW from baseline was −13.7% in the CagriSema group and −3.4% in the placebo group (estimated difference, −10.4% points; −11.2 to −9.5; P<0.001). Gastrointestinal AEs were more frequent in the cagrilintide–semaglutide group than in the placebo group (72.5% vs. 34.4%) |
| **2. Anticoagulants** | | | | |
| AZALEA-TIMI 71^59^  [NCT04755283](http://clinicaltrials.gov/show/NCT04755283) | Phase 2b, parallel-group, partially blind, R, active-controlled trial. 1287 with AF and CHA2DS2-VASc score ≥4 or CHA2DS2-VASc score of 3 with either planned concomitant use of antiplatelets or a CrCl ≤50 ml/min. Female: 44%. FU: 1.8 years | SC abelacimab (90 or 150 mg once monthly) administered in a blinded fashion vs oral rivaroxaban (20 mg od; 15 mg if CrCl ≤50 mL/min) administered in an open-label fashion | Major or clinically relevant non-major bleeding (according to ISTH criteria) | The incidence rate of the primary end point was 1.87 and 2.69 events per 100 person-years with 150-mg and 90-mg abelacimab, as compared with 8.14 events per 100 person-years with rivaroxaban (HR 0.33; 0.19-0.55; and 0.23; 0.13-0.42; both P<0.001). Major bleeding occurred in 2.3%, 1.9 and 7.2% and major GI bleeding in 0.5%, 0.5% and 4.1% of patients treated with abelacimab or rivaroxaban, respectively. |
| OCEANIC-AF^60^  NCT05643573 | Phase 3, R, DB trial. 14,810 with AF (at baseline or within the previous 12 months) and indication for indefinite treatment with an oral anticoagulant and had a CHA_2_DS_2_-VASc score ≥3 for men or ≥4 for women. Female: 35%. | Asundexian (50 mg od) or standard dose apixaban. | Whether asundexian was at least noninferior to apixaban for the prevention of stroke or systemic embolism | Asundexian was associated with a higher incidence of stroke or systemic embolism than treatment with apixaban in the period before the trial was stopped prematurely (1.3% vs. 0.4%; 3.79; 2.46-5.83). There were fewer major bleedings with apixaban than with asundexian (0.2% vs. 0.7%; 0.32; 0.18 -0.55). The incidence of any AE appeared to be similar in the two groups. |
| **3. Antihypertensive drugs** | | | | |
| ADVANCE-HTN^61^  NCT05769608 | Phase 2b, DB, R, PC trial. 285 uncontrolled hypertensives despite taking 2-5 antihypertensive drugs at baseline. Female: 38%. FU: 12 weeks | Lorundrostat 50 mg/day (stable dose) or 50 mg increased to 100 mg/day (dose-adjustment) if BP remained uncontrolled at 4 weeks vs. placebo | Placebo-adjusted change in 24-hour average SBP from baseline to week 12 in each lorundrostat group | The placebo-adjusted change in blood pressure was -7.9 mm Hg in the stable-dose group and -6.5 mm Hg in the dose-adjustment group and the placebo-adjusted change in 24-hour average SBP in the combined lorundrostat groups was -5.3 mm Hg. The percentage of participants with SBP <125 mm Hg was higher with lorundrostat than with placebo (41% vs. 18%; P<0.001). A serum potassium level >6.0 mmol/L occurred in 5-7% of patients treated with lorundrostat (none in the placebo group) |
| The Launch-HTN Randomized Clinical^62^  [NCT06153693](https://www.clinicaltrials.gov/study/NCT06153693?id=NCT06153693&rank=1) | Phase 3, R, DB trial. 1083 with treatment-resistant hypertension. Female: 46.9%. FU: 6 weeks | Lorundrostat (50 mg/d for 6 weeks followed by 100 mg/d 6 weeks if they met prespecified criteria), lorundrostat (50 mg/d) or dalily placebo for 12 weeks | Placebo-adjusted change in automated office SBP at week 6 for participants randomized to 50 mg | Mean change in office SBP at week 6 was −16.9 mm Hg for the pooled 50 mg of lorundrostat group vs −7.9 mm Hg for the placebo group (least-squares mean difference, −9.1 mm Hg; *P* <0.001). An office SBP <130 mm Hg was achieved in 44.1% of patients in the lorundrostat group vs 24.1% in the placebo group (*P*=0.003). Hyponatremia, hyperkalemia, and reduction in kidney function were reported more often with lorundrostat vs placebo |
| BaxHTN^63^ NCT06034743 | Phase 3, DB, R, PC trial. 796 with seated SBP between 140 and <170 mm Hg despite the treatment with 2 (uncontrolled hypertension) or ≥ 3 drugs (resistant hypertension), including a diuretic, for at least 4 weeks before screening. Female: 62.5%. FU: 12 weeks | Baxdrostat (1 or 2 mg daily), or placebo, in addition to background therapy | Change in the seated SBP from baseline to week 12, as assessed for each baxdrostat group, as compared with placebo | At week 12, the estimated difference from placebo (placebo-corrected difference) was –8.7 mm Hg (95% CI, –11.5 to –5.8) with 1-mg baxdrostat and –9.8 mm Hg (–12.6 to –7.0) with 2-mg baxdrostat (both P<0.001). The percentage of patients with a controlled seated SBP (<130 mm Hg) was 39.4% with 1-mg baxdrostat, 40.0% with 2-mg baxdrostat, and 18.7% with placebo (all P<0.001) |
| KARDIA-2^64^  NCT05103332 | Phase 2, R, P, DB trial. Patients received for at least 4 weeks with indapamide (2.5 mg), amlodipine (5 mg), or olmesartan (40 mg) od. Patients with 24-hour mean ambulatory SBP of 130 to 160 mm Hg were then randomized to zilebesiran (600 mg SC) or matching placebo. Female: 43.6%. FU: 6 months | A single SC dose of zilebesiran (600 mg) or placebo added to indapamide 2.5 mg, amlodipine 5 mg, or olmesartan 40 mg od | Difference between zilebesiran and placebo in change from baseline in 24-hour mean ambulatory SBP at 3 months | At 3 months, the least-squares mean difference between zilebesiran and placebo in 24-h mean ambulatory SBP was −12.1 mm Hg (−16.5 to −7.6; P<0.001) for the indapamide, −9.7 mm Hg (−12.9 to −6.6; P<0.001) for the amlodipine, and −4.5 mm Hg (95%CI, −8.2 to −0.8; P=0.02) for the olmesartan cohort. More patients on zilebesiran than on placebo experienced hyperkalemia (5.5% vs. 1.8%), hypotension (4.3% vs. 2.1%), and acute kidney failure (4.9% vs. 1.5%) |
| **4. Antiplatelets** | | | | |
| CELEBRATE^65^  NCT0482574 | Phase 3, DB, PC trial. 2467 with STEMI receive zalunfiban or placebo at first medical contact, either in the patient's home, the ambulance or the emergency department | Single SC injection of zalunfiban SC (0.11 and 0.13 mg/kg) or placebo | A hierarchical proportional odds model ranking 7 end points from worst to best: ACDh, stroke, recurrent MI, acute stent thrombosis, new-onset or rehospitalization for HF, larger infarct size, or no end point through 30 days | Zalunfiban improves the primary efficacy end point (aOR 0.79; 0.65-0.98; P=0.028). GUSTO severe bleeding was similar in both arms vs. placebo (1.2% vs. 0.8%; P=0.40), but GUSTO mild-moderate bleeding was increased (6.4% vs. 2.5%; P<0.001). Angiography showed faster coronary blood flow with zalunfiban vs. placebo (P=0.012). |
| **5. Cardiac myosin inhibitor in the treatment of HCM** | | | | |
| MAPLE-HCM^66^  NCT05767346 | Head-to-head, phase 3, DB, DD, R trial. 175 with HCM, resting obstruction (LVOT-G ≥30 mm Hg at rest) or latent LVOT-G obstruction ≥50 mm Hg after the Valsalva maneuver), symptoms and impaired functional capacity (NYHA class II-III, HF, KCCQ-CSS ≤90), and an age-/sex-predicted pVO2 <100%. Females: 42%. FU: 24 weeks | Aficamten (5 mg up to 20 mg daily) plus placebo or metoprolol (50 mg up to 200 mg daily) plus placebo. | The effect of aficamten vs. metoprolol on cardiac structure and function | Compared with metoprolol, aficamten significantly reduced resting LVOT-G (-30 mm HgP*<*0.001), Valsalva LVOT-G (-35 mm Hg; P<0.001) and left atrial volume index (-7.0 mL/m^2^; P*<*0.001); and improved E/e′ (lateral E/e′ -2.8 and septal -3.1) (all; *P<*0.001). Maximal wall thickness, LVEF, absolute LV global longitudinal strain, absolute global circumferential strain, mitral valve systolic anterior motion and mitral regurgitation significantly decreased in the aficamten group, with no significant change in resting cardiac output between groups |
| **6. Lipid-lowering drugs** | | | | |
| ALPACA^67^  NCT05565742 | Phase 2, R, DB, PC trial. 320, with elevated Lp(a) (median 253.9 nmol/L). Females: 43%. FU: 540 days | SC lepodisiran: 16, 96 or 400 mg at baseline and again at day 180; 400 mg at baseline and placebo at day 180; or SC placebo at baseline and at day 180. | Time-average percent change from baseline in serum Lp(a) concentration from day 60 to 180. | The placebo-adjusted time-averaged percent changes in Lp(a) levels from day 60 to day 180 were −40.8%, -75.2%, −93.5.1% in the 16 mg, 96 and pooled 400 mg dose groups, respectively. placebo-adjusted time-averaged percent reduction from baseline in Lp(a) levels at days 30 to 360 days was -41.2%, -77.2%, -94.8% and -88.5% in patients treated with 16 mg, 96 mg, 400-400 mg or 400 mg at baseline and placebo at day 180. Injection site reactions (8.1-11.6%) and liver transaminase elevation >3x the upper limit of normal (2.7-5.8%) were observed with lepodisiran |
| BROADWAY^68^ NCT05142722 | Phase 3, R, PC trial. 2530 with either heterozygous familial hypercholesterolemia or established ASCVD receiving maximum tolerated doses of lipid-lowering therapy. Female: 34%. FU: 1 year. | Obicetrapib (10 mg od) or matching placebo | Percent change in the LDL-C level from baseline to day 84 | Addition of obicetrapib to background therapy decreased LDL-C levels (-29.9% vs. +2.7% with placebo; between-group difference −32.6%; P<0.001) and the percentages of patients with LDL-C levels <40 mg/dL were 27.9% and 1.1%, respectively. LDL-C reduction persisted throughout one year, with a modest attenuation by day 365 (-24%). Obicetrapib also significantly reduced ApoB (-18.9%), non-HDL cholesterol (-29.4%), and Lp(a) (-33.5%) vs. placebo. The incidence of AEs was similar in the two groups. |
| CORALreef HeFH^69^  NCT05952869 | Phase 3, R trial. 303 adults with HeFH using lipid-lowering therapy (taking at least a moderate- or high-intensity statin) and either an LDL-C level ≥55 mg/dL and a history of major ASCVD or an LDL-C level ≥70 mg/dL without a history of major ASCVD. Female: 51%. FU: 52 weeks | Enlicitide (29 mg) or placebo of | Mean percentage change in LDL-C level at week 24 | The mean percentage change in LDL-C levels at weeks 24 and 52 were -58.2% and -55.3% in the enlicitide group vs 2.6% and 8.7% in the placebo group (between-group differences -59.4% and -61.5%; both P<.001). At week 24, the mean percentage change in non-HDL-C, ApoB anbd Lp(a) levels were -52.3%, -48.2% and -24.7% in the enlicitide group and 2.1%, 1.8% and -1.6%, respectively (all P<0.001). The incidence of AEs and SAEs was similar between groups |
| OCEAN(a)-DOSE Trial^70^  NCT04270760 | Phase 2, dose-finding, R, PC trial. 281 with ASCVD and Lp(a) levels >150 nmol/L (mean 260.3 nmol/L). Female 31.6%. FU: 36 weeks | Olpasiran 10 mg every 12 weeks (Q12W); 75 mg, Q12W; 225 mg, Q12W; or 225 mg, administered every 24 weeks (Q24W) vs. placebo | Placebo-adjusted change  in oxidized phospholipids (OxPL)-apoB from baseline to wee | The placebo-adjusted mean percentage change in OxPL-apoB from baseline to week 36 was −51.6%, −89.7%, −92.3%, and −93.7% in the olpasiran groups (all P<0.001 for all) and the effects were maintained to week 48. However, olpasiran did not significantly impact hs-CRP or hs-IL-6 vs. placebo |
| PROLONG-ANG3^71^  NCT05256654 | Phase 2, DB, PA, R, PC trial. 205 with mixed dyslipidemia (fasting TG 1.69-5.64 mmol/L, LDL-C ≥1.81 mmol/L, and non-HDL-C ≥3.36 mmol/L) and BMI 18.5-40.0 kg/m^2,^ on moderate/high-intensity statins. Females: 54%. FU: 270 days. | SC solbinsiran: 100 mg, 400 mg or 800 mg, or placebo on days 0 and 90 | Placebo-adjusted change in apoB levels from baseline to day 180 | Solbinsiran 100, 400 and 800 mg reduced apoB levels by -2.8%, -14.3%, and -8.3%, and ANGPTL-3 levels by –54.3% to–76.6% (all p<0.0001), respectively. At 400 mg reduced non-HDL-C (25.5%), LDL-C (16.8%), TG (50.3%), ApoB (14.3%) and VLDL-C levels (50.1%) and increased HDL-C (16.4%). The most common AEs were gastroenteritis/nasopharyngitis (4%) and hypertension, urinary tract infection, influenza and local site reactions (3%) |
| PURSUIT^72^  NCT06173570 | Phase 2, R, DB, PC trial. 428 with hypercholesterolemia (LDL-C levels ≥70 but <190 mg/dL) and TG <400 mg/dL on stable dose of moderate/high-intensity statins. Females: 48%. FU: 12 weeks | Oral Laroprovstat (AZD0780, 1, 3, 10, or 30 mg or matching placebo o.d. for 12 weeks | Percent of change of LDL-C from baseline to week 12 | At week 12, placebo-adjusted difference in LDL-C levels with AZD0780 were -35.3%, -37.9%, -45.2% and -50.7% (all P<0.001), respectively. The proportion of patients with LDL-C levels <70 mg/dL dose-dependently increased from 56.8% to 82.2% with laroprovstat(13% with placebo). AEs were similar in both arms |
| REMAIN-3^73^  NCT04844125 | Phase 3, R, DB, PC trial. 143 with HeFH on stable lipid-lowering therapy for ≥28 days (fasting LDL-C ≥2.6 mmol/L or ≥1.8 mmol/L for those with a history of ASCVD). Female: 52%. FU: 12 weeks | SC recaticimab at 150 mg or matching placebo Q4W for 12 weeks | Oercentage change in LDL-C from baseline to Week 12. | Recaticimab was superior to placebo to decrease the mean percentage change in LDL-C from baseline (−54.4%; −57.9 to −50.8% vs. −4.5%; −9.4 to 0.3%; P<0.0001) and in improving non-HDL-C, apoB, and lipoprotein a. The most common treatment-related AEs were injection site reactions (8.4% vs. 0%) and increased blood creatine phosphokinase (5.3% vs. 2.1%). |
| TANDEM^74^  NCT06005597 | Phase 3, R, DB, PC trial. 407 with pre-existing or high risk for ASVCD or HeFH with LDL-C levels ≥1.8 mmol/L (70 mg/dL) despite maximally tolerated lipid-lowering therapy excluding ezetimibe or having statin intolerance. Females: 43.5%. FU: 84 days | Oral obicetrapib 10 mg plus ezetimibe 10 mg FDC, obicetrapib 10 mg, ezetimibe 10 mg monotherapy, or placebo | Percent of changes in the FDC group compared with placebo, ezetimibe monotherapy, and obicetrapib monotherapy, and the placebo-adjusted change in the obicetrapib  monotherapy group. | At day 84, the difference in the reduction of LDL-C levels in the FDC group were –48·6% vs. placebo, –27·9% vs. versus ezetimibe monotherapy, and –16·8% vs. obicetrapib monotherapy (all P<0.001). Obicetrapib monotherapy decreased LDL-C by 31.9% vs. placebo (P<0·0001). AEs rates were similar in the FDC, obicetrapib and ezetimibe groups (51-54%) and lowest with placebo (37%) |

***** In alphabetic order ** Acronyms of the trials are summarized in **Supplemental Table 7.**

Abbreviations. ACD: all-cause death. AEs: adverse events. aOR: adjusted odds ratio. ApoB: apolipoprotein B. ASCVD: atherosclerotic cardiovascular disease. BMI: body mass index. DB: double blind. DD: double dummy. FDC: fixed-dose combination. FU: follow-up. GI: gastrointestinal. HbA1C: glycated haemoglobin. HCM: hypertrophic cardiomyopathy. HDL-C: high-density lipoprotein cholesterol. HF: heart failure. HR: hazard ratio. HeHF: heterozygous familial hypercholesterolaemia. LDL-C: low-density lipoprotein cholesterol. Lp(a): lipoprotein (a). LVOT: left ventricular outflow tract. MI: myocardial infarction. NCT: ClinicalTrials.gov identifier. Od: once daily. NT-proBNP: N-terminal pro-B-type natriuretic peptide. NYHA: New York Heart Association. P: prospective. PA: parallel assignment. PC: placebo controlled. Q4W/Q8W: every 4 or 8 weeks. pVO2: peak oxygen uptake. R: randomized. SAEs: serious adverse events. SBP: systolic blood pressure. SC: subcutaneous.: triglycerides. STEMI: ST-segment elevation MI. T2D: type 2 diabetes mellitus. VLDL-C: very low-density lipoprotein cholesterol.

### **Supplemental Table 6. New drugs in phase 2 and 3 of clinical development**

| **Pharmacological class** | **Drug** | **Mechanism of action** | **Clinical trials (Acronym, NCT Identifier Number)** |
| --- | --- | --- | --- |
| Antihypertensive drugs | Baxdrostat | Highly selective aldosterone synthase inhibitors | Resistant hypertension: BaxHTN, NCT06034743; BaxAsia, NCT06344104. Primary aldosteronism: Bax24, NCT06168409. Plus dapagliflozin in CKD and high BP: BaxDuo-Pacific, NCT06742723; NCT06268873. With dapagliflozin in HF: Prevent-HF, NCT06677060 |
|  | Lorundrostat |  | Hypertension: NCT05968430. Hypertension and OSA: NCT06785454 |
|  | Vicadrostat |  | Hypertension, T2D and CKD: NCT07064473. Plus empagliflozin in HF: NCT06424288; NCT06935370. Plus empagliflozin in CKD: EASi-KIDNEY, NCT06531824; NCT06926660 |
|  | Zilebesiran | siRNA targeting hepatic ANG mRNA expression | High CV risk and hypertension not adequately controlled: KARDIA-3, NCT06272487; ZENITH, NCT07181109 |
| Antiarrhythmics | CRD-4730 | CaMKII inhibitor | Catecholaminergic polymorphic ventricular tachycardia: NCT06658899 |
| Anticoagulants  (Fact XIa inhibitors) | Abelacimab (MAA868) | mAb that binds to the catalytic domain of FXI and prevents its activation | High-risk patients with AF unsuitable for OAC: LILAC-TIMI 76, NCT05712200. Treatment of cancer-associated VTE: ASTER, NCT05171049; MAGNOLIA, NCT05171075 |
|  | Asundexian | Small molecule that targets the active site of FXIa and blocks its activity | AF ineligible for OAC: OCEANIC-AFI, CT06124612. Secondary stroke prevention: OCEANIC-STROKE, NCT05686070. |
|  | Milvexian | Small molecule, orally active, inhibitor of factor XIa | Post-MI: LIBREXIA-ACS, NCT05754957. Secondary stroke prevention: LIBREXIA-STROKE, NCT05702034). AF vs. apixaban: LIBREXIA-AF, NCT05757869 |
|  | REGN9933 | mAb that binds and inhibits FXIa activation | Peripherally inserted central catheter: ROXI-CATH, NCT06299111. AF: ROXI-ATLAS, NCT07175428 |
| Anti-obesity drugs | CagriSema | Combination of cagrilintide, a long-acting amylin analog, with semaglutide | Obesity: RENEW 1, NCT07220642; REDEFINE 1, NCT05567796; REDEFINE 6, NCT05996848; NCT06780449; NCT06388187; NCT06131437; NCT06207877. Obesity and T2D: RENEW 2, NCT07220759; REFINE 2, NCT05394519; NCT06221969. T2D plus semaglutide: NCT06716307, NCT07184086; NCT06267092; NCT07011667. T2D plus metformin: REIMAGINE 2, NCT06065540; REIMAGINE 5, NCT06534411. T2D plus metformin ± a SGLTi: NCT06221969. Obesity, T2D and CKD: NCT06131372. T2D: REIMAGINE 1, NCT06323174; REIMAGINE 3, NCT06323161; NCT06403761. CVD: REDEFINE 3, NCT05669755. T2D and painful diabetic peripheral neuropathy: NCT06797869 |
|  | Maridebart cafraglutide | Peptide–antibody conjugate acting as GLP-1 receptor agonist and GIP receptor antagonist | T2D: NCT06660173, NCT07160257. T2D with obesity or overweight: MARITIME-1, NCT06858839; MARITIME-2, NCT06858878. Obesity: MARITIME-3-J, NCT06987695. Obesity and HFp/mrEF: MARITIME-HF, NCT07037459. Obesity and ASCVD: MARITIME-CV, NCT07037433 |
|  | Mazdutide | GLP-1 and glucagon receptor dual agonist | HFpEF or HfmrEF combined with obesity: NCT06862908. OSA and obesity: NCT06931028. T2D and obesity:  NCT06184568, NCT06143956 |
|  | Orforglipron | GLP-1 receptor agonist | T2D: ACHIEVE-2, NCT06192108; ACHIEVE-5, CT06109311. Hypertension and obesity/overweight: ATTAIN-Hypertension, NCT06948422, NCT06948435, NCT06952530. Obesity or overweight and knee osteoarthritis: NCT07153471**.** Obesity/overweight with/without T2D: NCT06993792, NCT06824051, or weight-related comorbidity: ATTAIN- MAINTAIN, NCT06584916, NCT06972459, NCT05869903, CT06672939. OSA: ATTAIN-OSA, NCT06649045 |
|  | Pemvidutide | GLP-1 and glucagon receptor agonist | Obesity or Overweight: RECLAIM STUDY, NCT06987513 |
|  | Retatrutride | Agonist of GIP, GLP-1 and glucagon receptors | Overweight/obesity: TRIUMPH-1, NCT05929066; TRIUMPH-5, NCT06662383; TRIUMPH-6, NCT06859268. T2D: NCT06982846, NCT06982859; NCT06354660. Overweight/obesity + T2D: TRIUMPH-2, NCT05929079. Overweight/obesity + CKD ± T2D: NCT05936151; TRANSCEND-T2D-1, 2, 3 (NCT06354660, NCT06260722, NCT06297603). Overweight/obesity + knee osteoarthritis: TRIUMPH-4, NCT05931367. Overweight/obesity and chronic low back pain: TRIUMPH-7, NCT07035093. Obesity + CVD: TRIUMPH-3, NCT05882045. CV and kidney outcomes in patients with obesity: TRIUMPH-OUTCOMES, NCT06383390. |
|  | Survodutide | GLP-1 and glucagon receptor agonist | Overweight/obesity: SYNCHRONIZE™JP: NCT06176365; SYNCHRONIZE™ - CVOT, NCT06077864; NCT06214741, NCT06200467. Kidney disease: ARTIST-CKD, NCT07206290 |
| Antiplatelets | Glenzocimab | Humanized Fab fragment 9O12 against the extracellular domains of GPVI | For reperfusion in the setting of endovascular therapy for brain infarct: GREEN, NCT05559398. Anterior stroke with large ischemic core eligible for endovascular therapy: GALICE, NCT06437431. STEMI: LIBERATE, ISRCTN15443962 |
|  | Ralinepag | Selective prostacyclin receptor agonist | PAH: NCT03683186, NCT03626688 |
|  | Selatogrel | P2Y12 receptor antagonist | AMI: SOS-AMI, NCT04957719 |
|  | Zalunfiban | Glycoprotein IIb/IIIa inhibitor | ST-elevation MI: CELEBRATE, NCT04825743 |
| Dislypidaemia | DR10624 | Fc fusion protein tri-agonist of GLP-1, glucagon and FGF21 receptors | Severe HT: CT06555640. HT and carotid atherosclerotic plaque: NCT07050134 |
|  | Enlicitide decanoate | Oral PCSK9 Inhibitor | Hypercholesterolemia: CORAreef-extension, NCT06492291. HeFH: NCT07058077. MACE: CORALreef Outcomes, NCT06008756 |
|  | Laroprovstat | Small molecule inhibitor of PCSK9 | HeFH: AZURE-HeFH¸ NCT07000136. Clinical ASCVD or at Risk for a first ASCVD event: AZURE-LDL, NCT07000123; AZURE-Outcomes, NCT07000357. Plus rosuvastatin in patients with dyslipidaemia: LAZURE (NCT07218900) |
|  | Lepodisiran | GalNAC-conjugated siRNA that inhibits the hepatic synthesis of lp(a) | Elevated Lp(a): ACCLAIM-Lp(a), NCT06292013 |
|  | Lerodalcibep | Recombinant fusion protein of a PCSK9-binding domain (adnectin) and human serum albumin | Hypercholesterolemia: NCT06568471. HeFH: LiberateKids, NCT07102511. Patients with CVD on Statins: LIBerate-CVD, NCT04797247. CVD or at high-risk for CVD: LIBerate-HR, NCT04806893; HoFH, HeFH, and high-risk CVD patients requiring further LDL-reduction: LIBerate-OLE, NCT04798430 |
|  | Muvalaplin | Oral small molecule that inhibits the apo(a)-apo B_100_ interaction | Elevated Lipoprotein(a) at high risk for CV events: MOVE-Lp(a): CT07157774 |
|  | Obicetrapib | Selective cholesteryl ester transfer protein inhibitor | Elevated Lp(a) Levels: VINCENT, NCT06496243; NCT05972278. Plus statins: ROSE, NCT04753606. Plus ezetimibe: RUBENS, NCT07219602. ASCVD: PREVAIL, NCT05202509 |
|  | Olezarsen | Ligand conjugated ASO targeting apoC-III | Severe HT: CORE-OLE, NCT05681351. FCS: NCT05130450. FCS treated with volanesorsen: NCT05185843 |
|  | Olpasiran | siRNA that prevents assembly of Lp(a) | ASCVD: OCEAN(a), NCT05581303. Prevent first MACE; OCEAN(a)-PreEvent,  NCT07136012 |
|  | Pelacarsen | ASO targeting the mRNA transcribed from the *LPA* gene | Elevated Lp(a) + ASCVD: Lp(a)HORIZON, NCT04023552; OLE, NCT05900141. Elevated Lp(a) and established ASCVD: DD-VANTAGE, NCT06813911; NCT06875973; NCT06267560. Calcific aortic valve stenosis: NCT05646381 |
|  | Plozasiran | siRNA targeting the hepatic production of apo C-III | Mixed dyslipidemia: NCT05413135. HT: NCT06347133; SHASTA-3, NCT06347003; SHASTA-4, NCT06347016; SHASTA-10, NCT06822790; MUIR-3, NCT06347133. FCS: NCT05902598 |
|  | Recaticimab | PCSK9 inhibitor | ACS: ELITE-ACS, NCT06738758. Intracranial Atherosclerotic Symptomatic Stenosis: PICASSO, NCT07119918; PISTIAS-2, NCT06902740. Acute Ischemic Stroke: EAST-LDL, NCT07002476 |
|  | SHR-1918 | Fully humanized mAb against ANGPTL-3 | Hypercholesterolaemia: NCT07133815, NCT06471218. HoFH: NCT07133815, NCT06723652, NCT06723652 |
|  | Tafolecimab | Fully human IgG2 mAb that specifically binds to PCSK9 | Before percutaneous coronary intervention in AMI: IMPROVE-AMI, NCT06683131; NCT06096909, NCT05457582. Calcific aortic valve stenosis: NCT04968509 |
|  | Zerlasiran | siRNA to inhibit Lp(a) production | High risk for atherosclerotic CV events and elevated of Lp(a): NCT05537571 |
|  | Zodasiran | siRNA targeting *ANGPTL3* gene expression in the liver | HoFH: YOSEMITE, CT07037771; NCT06712771 |
| ET-1 receptor antagonists | Zibotentan | Selective ET-A antagonist | CKD and high proteinuria: ZODIAC, NCT06942910; ZENITH high proteinuria, NCT06087835 |
| Fabri disease | Lucerastat | Glucosylceramide synthase inhibitor | Fabry disease: MODIFY, NCT03425539; RWE-FABRY,  NCT06303466; CARAT, NCT05280548; NCT03737214; NCT06906367, NCT04252066. With amenable GLA variants: NCT04020055, NCT06904261 |
|  | Migalastat | Chaperone that stabilizes misfolded, unstable α-galactosidase A enzymes | Fabry subjects with amenable *GLA* variants (NCT06904261, NCT01218659) and renal disease: NCT04020055. Women With Fabry Disease and Their Infants During Pregnancy and Breastfeeding: NCT04252066 |
|  | Venglustat | Glucosylceramide synthase inhibitor | Fabry disease: PERIDOT, NCT05206773. Gaucher Disease Type 3; LEAP2MONO, NCT05222906 |
| Friedreich’s ataxia cardiomyopathy | LX2006 | AAV-based gene therapy designed to deliver a functional frataxin gene | Friedreich’s ataxia: NCT05445323 |
| Hypertrophic cardiomyopathy | Aficamten | Cardiac myosin inhibitor | oHCM: NCT06116968; FOREST-HCM, NCT04848506; CAMELLIA-HCM, NCT07023341; CEDAR-HCM, NCT06412666. nHCM: ACACIA-HCM, NCT06081894 |
|  | BMS-986435 | Cardiac myosin modulator | oHCM: NCT05667493; HFpEF: AURORA-HFpEF, NCT06122779. |
|  | Ninerafaxstat | Partial fatty acid oxidation (pFOX) inhibitor | noHCM: FORTITUDE-HCM),  NCT07023614 |
|  | TN-201 | AVV9-based gene therapy designed to deliver the *MYBPC3* gene | Symptomatic MYBPC3 mutation-associated HCM: MyPEAK-1, NCT05836259; NCT05112237 |
| Heart failure | Balcinrenone | Nonsteroidal, selective MRA | CKD: NCT05884866 (plus dapagliflozin). HF with CKD: BalanceD-HF, NCT06307652. Balcinrenone ± dapagliflozin in patients with CKD: DapaBalci-Leap, NCT05884866 |
|  | CDR132L | Specific ASO, miR-132 inhibitor | HFpEF and LVH: 8212-Preserved, NCT06979362. HFmr/rEF: 8232-Reduced, NCT06979375. HFrEF post-MI: HF-REVERT, EudraCT Number: 2021-006040-27 |
|  | Ziltivekimab | Fully human mAb targeting the interleukin-6 ligand | Inflammation: SPIDER, NCT NCT06263244. ASCVD, CKD and inflammation: ZEUS trial, NCT05021835. HF + inflammation: ATHENA, NCT06200207; HERMES, NCT05636176. AMI: ARTEMIS, NCT06118281 |
| Pulmonary hypertension | Sotatercept | Chimeric protein containing the extracellular domain of the activin receptor 2A fused to the Fc domain of human IgG1 | PAH: RECOMPENSE, NCT06658522; LIGHTRAY, NCT06664801; LIGHTRAY EXT, NCT06925750; MOONBEAM, NCT05587712; SOTERIA, NCT04796337; NCT07140484; NCT06409026; NCT06751082. Cpc-PH due to HFpEF: CADENCE, NCT04945460. Central cardiopulmonary performance and peripheral oxygen transport during exercise in PAH: NCT06409026 |
| Systemic amyloidosis | AT-02 | Pan-amyloid-binding humanized IgG1-peptide fusion | Systemic smyloidosis: AT02-001, NCT05521022; NCT05951049; |
| Transthyretin-mediated amyloid cardiomyopathy | Coramitug | Humanized mAb designed to target and clear the non-native transthyretin aggregates (misTTR) | HF due to transthyretin amyloid (ATTR) amyloidosis: CLEOPATTRA, NCT07207811 |
|  | Eplontersen | Ligand-conjugated ASO to inhibit the production of hepatic TTR | ATTR-CM: NCT05667493; EPIC-ATTR, NCT06194825; CARDIO-TTRansform, NCT04136171; |
|  | NI006 | Antibody producing depletion of cardiac amyloid load | DepleTTR-CM, NCT06183931 |
|  | NTLA-2001 | Knocking out the TTR gene | ATTR-CM: NCT04601051, NCT06672237, NCT06128629; NCT05697861 |

Abbreviations. AAV9: adeno-associated virus serotype 9. AF: atrial fibrillation. AMI: acute myocardial infarction. *ANG*: gene encoding angiotensinogen. *ANGPTL3*: gene encoding angiopoietin-like protein 3. Apo: apolipoprotein. ASCVD: atherosclerotic cardiovascular disease. ASO: antisense oligonucleotide. ATTR-CM: transthyretin-mediated amyloid cardiomyopathy. AVV9: adeno-associated virus. BP: blood pressure. CaMKII: calcium/calmodulin dependent protein kinase II inhibitor. CKD: chronic kidney disease. Cpc-PH: combined post-capillary and pre-capillary pulmonary hypertension. CV: cardiovascular. CVD: cardiovascular disease. FCS: familial chylomicronemia syndrome. FGF21R: fibroblast growth factor 21 receptor. FXI: coagulation factor XI. GalNAc: *N*-acetyl-galactosamine. GIP: glucose-dependent insulinotropic polypeptide. GLP1: glucagon-like peptide 1. GP: glycoprotein. GPVI: glycoprotein IV. HCM: hypertrophic cardiomyopathy. HeFH: heterozygous familial hypercholesterolemia. HF: heart failure. HFpEF: heart failure with preserved ejection fraction. HFrEF: heart failure with reduced ejection fraction. HoFH: homozygous familial hypercholesterolemia. HT: hypertriglyceridemia. Ig: immunoglobulin. Lp(a): lipoprotein (a). *LPA*: lipoprotein(a) gene. LVH: left ventricular hypertrophy. mAb: monoclonal antibody. MACE: major cardiovascular events. MI: myocardial infarction. MR: mineralocorticoid receptor. MRA: MR antagonist. mRNA: messenger RNA. MYBPC3: gene encoding myosin binding protein C3. NCT: National Clinical Trial. noHCM: nonobstructive hypertrophic cardiomyopathy. OAC: oral anticoagulant. oHCM: obstructive hypertrophic cardiomyopathy. OSA: obstructive sleep apnea. PAH: pulmonary hypertension. PCSK9: proprotein convertase subtilisin/kexin type 9. siRNA: small interfering RNA. STEMI: ST elevation myocardial infarction. T2D: type 2 diabetes. TTR: transthyretin. VTE: venous thromboembolism.

**Supplemental Table 7. Acronyms of ongoing clinical trials**

| **Acronym** | **Title** |
| --- | --- |
| AβYSS | Assessment of β-blocker interruption one Year after an uncomplicated myocardial infarction on Safety and Symptomatic cardiac events requiring hospitalization |
| ACHIEVE-1 | A Study of Orforglipron (LY3502970) in Adult Participants With Type 2 Diabetes and Inadequate Glycemic Control With Diet and Exercise Alone |
| ACTRN12613000065796 | Anti-fibrotic role of eplerenone on diffuse myocardial fibrosis and diastolic function in patients with hypertrophic cardiomyopathy |
| ADAPT AF-DES | Appropriate Duration of Antiplatelet and Thrombotic Strategy after 12 Months in Patients with Atrial Fibrillation Treated with Drug-Eluting Stents |
| ADJUST-T1D | ADJUnct Semaglutide Treatment in Type 1 Diabetes |
| ADVANCE-HTN | Subjects With Uncontrolled Hypertension on a Standardized Antihypertensive Medication Regimen |
| AFIRE | Atrial Fibrillation and Ischemic Events With Rivaroxaban in Patients With Stable Coronary Artery Disease |
| ALONE AF | AnticoaguLation ONE Year After Ablation of Atrial Fibrillation in Patients With Atrial Fibrillation |
| ALPACA | A Study of LY3819469 in Participants With Elevated Lipoprotein(a) [Lp(a)] |
| ANGEL-TNK | Intra-arterial Recombinant Human TNK Tissue-type Plasminogen Activator (rhTNK-tPA) Thrombolysis for Acute Large Vascular Occlusion After Successful Mechanical Thrombectomy Recanalization |
| ANNEXA-I | Trial of Andexanet Alfa in ICrH Patients Receiving an Oral FXa Inhibitor |
| API-CAT | API-CAT STUDY for APIxaban Cancer Associated Thrombosis |
| AQUATIC | Assessment of Quitting versus Using pirin Therapy in Patients with Stabilized Coronary Artery Disease after Stenting Who Require Long-Term Oral Anticoagulation |
| ARTESIA | Apixaban for the Reduction of Thrombo-Embolism in Patients With Device-Detected Sub-Clinical Atrial Fibrillation |
| ASSET-IT | Advancing Stroke Safety and Efficacy through Early Tirofiban Ad­ministration after Intravenous Thrombolysis |
| ATTAIN-1 | A Study of Orforglipron (LY3502970) in Adult Participants With Obesity or Overweight With Weight-Related Comorbidities |
| ATTRibute-CM | Efficacy and Safety of AG10 in Subjects With Transthyretin Amyloid Cardiomyopathy |
| AZALEA–TIMI 71 | Safety and Tolerability of Abelacimab vs. Rivaroxaban in Patients with Atrial Fibrillation–Thrombolysis in Myocardial Infarction 71 |
| BaxHTN | A Study to Investigate the Efficacy and Safety of Baxdrostat in Participants With Uncontrolled Hypertension on Two or More Medications Including Participants With Resistant Hypertension |
| BETAMI–DANBLOCK | BETAMI: Betablocker Treatment After Acute Myocardial Infarction in Patients Without Reduced Left Ventricular Systolic Function  DANBLOCK: Danish Trial of Beta Blocker Treatment After Myocardial Infarction Without Reduced Ejection Fraction |
| BROADWAY | Randomized Study to Evaluate the Effect of Obicetrapib on Top of Maximum Tolerated Lipid-Modifying Therapies |
| BRIDGE-TNK | Randomized Trial of Thrombectomy with versus without Recombinant Human Tenecteplase (TNK) Tissue Plasminogen Activator in Stroke |
| CAVIAR | Cardiac Allograft Vasculopathy Inhibition with Alirocumab |
| CLEAR SYNERGY | Colchicine and Spironolactone in Patients with MI /​ SYNERGY Stent Registry |
| COCOMO-ACS | Colchicine for Coronary Plaque Modification in Acute Coronary Syndrome |
| CONFIDENCE | A Study to Learn How Well the Treatment Combination of Finerenone and Empagliflozin Works and How Safe it is Compared to Each Treatment Alone in Adult Participants With Long-term Kidney Disease (Chronic Kidney Disease) and Type 2 Diabetes |
| CORALreef HeFH | A Study of Enlicitide Decanoate (MK-0616 Oral PCSK9 Inhibitor) in Adults With Heterozygous Familial Hypercholesterolemia |
| CORE-TIMI 72a | A Study of Olezarsen (ISIS 678354) Administered to Participants With Severe Hypertriglyceridemia |
| CORE-TIMI 72b | A Study of Olezarsen Administered Subcutaneously to Participants With Severe Hypertriglyceridemia |
| DAPA-TAVI | Dapagliflozin After Transcatheter Aortic Valve Implantation |
| DECODE-CKD | Effects of Dapagliflozin on EchOcardiographic Measures of CarDiac StructurE and Function in Patients with CKD |
| DIGIT | Digitoxin to Improve Outcomes in Patients with Advanced Chronic Heart Failure |
| DOAC-CVT | Direct oral anticoagulants versus vitamin K antagonists for cerebral venous thrombosis |
| DUAL-ACS | Duration of Dual Anti-Platelet Therapy |
| EMPA-KIDNEY | The Study of Heart and Kidney Protection With Empagliflozin |
| ESSENCE-TIMI 73b | A Study of Olezarsen (ISIS 678354) in Participants With Hypertriglyceridemia and Atherosclerotic Cardiovascular Disease, or With Severe Hypertriglyceridemia |
| EXPECTS | The Extending the Time Window for Thrombolysis in Posterior Circulation Stroke without Early CT Signs |
| FARES-II | Active-control Randomized Trial Comparing 4-factor Prothrombin Complex Concentrate With Frozen Plasma in Cardiac Surgery |
| FINEARTS-HF | Study to Evaluate the Efficacy (Effect on Disease) and Safety of Finerenone in Participants With Heart Failure and Left Ventricular Ejection Fraction (Proportion of Blood Expelled Per Heart Stroke) Greater or Equal to 40% |
| FINE-HEART | An Integrated Pooled Analysis of Finerenone across 3 Phase III Trials of Heart Failure and Chronic Kidney Disease and Type 2 Diabetes |
| FOURIER | Further Cardiovascular Outcomes Research with PCSK9 Inhibition in Subjects with Elevated Risk |
| GEIST | The German Italian Spanish Takotsubo (GEIST) Registry |
| GLORY-1 | A Randomized, Double-blind, Placebo-controlled Phase III Study Evaluating the Efficacy and Safety of IBI362 in Chinese Participants With Obesity or Overweight |
| HELIOS-B | A Study to Evaluate Vutrisiran in Patients With Transthyretin Amyloidosis With Cardiomyopathy |
| HI-PRO | Extended-Duration Low-Intensity Apixaban to Prevent Recurrence in High-Risk Patients With Provoked Venous Thromboembolism |
| HOST-BR | HOST – DAPT Duration According the Bleeding Risk |
| HYPERION | Study of Sotatercept in Newly Diagnosed Intermediate- and High-Risk PAH Participants (MK-7962-005/​A011-13) |
| KARDIA-2 | Zilebesiran as Add-on Therapy in Patients With Hypertension Not Adequately Controlled by a Standard of Care Antihypertensive Medication |
| KARDIA-3 | Zilebesiran as Add-on Therapy in Patients With Hypertension Not Adequately Controlled by a Standard of Care Antihypertensive Medication |
| LIBerate-HeHF | Randomized, Double-Blind, Placebo-Controlled, Phase 3 Study to Evaluate the Long-Term Efficacy and Safety of LIB003 in Heterozygous Familial Hypercholesterolemia Patients on Stable Lipid-Lowering Therapy Requiring Additional Low-Density Lipoprotein Cholesterol Reduction |
| LIBerate-HR | Study of Long-Term Efficacy and Safety of LIB003 in CVD or High Risk for CVD Patients Needing Further LDL-C Reduction |
| MAPLE-HCM | Metoprolol versus Aficamten in Patients with Left Ventricular Outflow Tract Obstruction on Exercise Capacity in HCM |
| NCT03080935 | Fourier Open-label Extension Study in Subjects With Clinically Evident Cardiovascular Disease in Selected European Countries |
| NCT05669599 | Dose-ranging Study to Evaluate the Efficacy, Safety, and Tolerability of AMG 133 in Adult Subjects With Overweight or Obesity, With or Without Type 2 Diabetes Mellitus |
| [NCT06153693](https://www.clinicaltrials.gov/study/NCT06153693?id=NCT06153693&rank=1) | Efficacy and Safety of Lorundrostat in Subjects With Uncontrolled and Resistant Hypertension |
| NEO-MINDSET | Percutaneous Coronary Intervention Followed by Monotherapy Instead of Dual Antiplatelet Therapy in the Setting of Acute Coronary Syndromes |
| NEURO-TTRansform | A Study to Evaluate the Efficacy and Safety of Eplontersen in Participants With Hereditary Transthyretin-Mediated Amyloid Polyneuropathy |
| NODE-303 | Safety Study of Etripamil Nasal Spray for Patients With Paroxysmal Supraventricular Tachycardia |
| OASIS 4 | Research Study Looking at How Well Semaglutide Tablets Taken Once Daily Work in People Who Have a Body Weight Above the Healthy Range |
| OCEANIC-AF | Oral Factor 11a Inhibitor Asundexian as Novel Antithrombotic-Atrial Fibrillation |
| ODYSSEY-HCM | A Study of Mavacamten in Non-Obstructive Hypertrophic Cardiomyopathy |
| ORION-13 | Study to Evaluate Efficacy and Safety of Inclisiran in Adolescents With Homozygous Familial Hypercholesterolemia |
| PALISADE | Study of ARO-APOC3 (Plozasiran) in Adults With Familial Chylomicronemia Syndrome (FCS) |
| POST-TNK | Safety and efficacy of adjunctive intra-arterial Tenecteplase after successful endovascular thrombectomy in patients with large vessel occlusion stroke |
| PRESTIGE-AF | PREvention of Stroke in Intracerebral aemorrhage Survivors With Atrial Fibrillation |
| PROLONG-ANG3 | A Study of LY3561774 in Participants With Mixed Dyslipidemia |
| PURSUIT | A Study to Assess the Efficacy, Safety and Tolerability of Different Doses of AZD0780 in Patients With Dyslipidemia |
| RAPID (NODE-301) | Efficacy and Safety of Etripamil for the Termination of Spontaneous Paroxysmal Supraventricular Tachycardia (PSVT) |
| REBOOT | We conducted the Treatment with Beta-Blockers after Myocardial Infarction without Reduced Ejection Fraction |
| REDEFINE 1 | A Research Study to See How Well CagriSema Helps People With Excess Body Weight Lose Weight |
| REDEFINE 2 | A Research Study to See How Well CagriSema Helps People With Type 2 Diabetes and Excess Body Weight Lose Weight |
| REMAIN-3 | SHR-1209 Treatment Efficacy and Safety of the Patients With Hypercholesterolemia Ⅲ Period Clinical Research |
| RETREAT-FRAIL | Impact of Reducing Antihypertensive Treatment on Mortality in Frail Subjects With Low Systolic Blood Pressure (SBP) |
| RIVAWAR | Rivaroxaban in Left Ventricular Thrombus |
| SEQUOIA-HCM | Aficamten vs Placebo in Adults With Symptomatic Obstructive Hypertrophic Cardiomyopathy |
| SMART-CHOICE3 | SMart Angioplasty Research Team: CHoice of Optimal Anti-Thrombotic Strategy in Patients Undergoing Implantation of Coronary Drug-Eluting Stents 3 |
| SOGALDI-PEF | Sodium-Glucose cotransporter 2 inhibitor with and without an ALDosterone AntagonIst for heart failure with preserved ejection fraction |
| SOUL | Semaglutide Cardiovascular Outcomes Trial |
| START | Optimal Delay Time to Initiate Anticoagulation After Ischemic Stroke in Atrial Fibrillation |
| STEP | Strategy of Blood Pressure Intervention in the Elderly Hypertensive Patients |
| STOP-CA | Statins TO Prevent the Cardiotoxicity From Anthracyclines |
| STRIDE | A Research Study to Compare a Medicine Called Semaglutide Against Placebo in People With Peripheral Arterial Disease and Type 2 Diabetes |
| SUMMIT | A Study of Tirzepatide (LY3298176) in Participants With Heart Failure With Preserved Ejection Fraction (HFpEF) and Obesity |
| SURMOUNT-1 | A Study of Tirzepatide (LY3298176) in Participants With Obesity or Overweight |
| SURMOUNT-5 | A Study of Tirzepatide (LY3298176) in Participants With Obesity or Overweight With Weight Related Comorbidities |
| SURMOUNT-OSA | Obstructive Sleep Apnea Master Protocol GPIF: A Study of Tirzepatide (LY3298176) in Participants With Obstructive Sleep Apnea |
| TACSI | Dual Antiplatelet Therapy with Ti­cagrelor and Acetylsalicylic Acid [ASA] vs. ASA Only after Isolated Coronary Artery Bypass Graft­ing in Patients with Acute Coronary Syndrome) |
| TADCLOT | Twice-A-Day CLOpidogrel vs Ticagrelor |
| TAILORED-CHIP | TAILored Versus COnventional AntithRombotic StratEgy IntenDed for Complex HIgh-Risk PCI |
| TANDEM | Study of Obicetrapib & Ezetimibe Fixed Dose Combination on Top of Maximum Tolerated Lipid-Modifying Therapies |
| TARGET-FIRST | Evaluation of a Modified Anti-Platelet Therapy Associated With Low-dose DES Firehawk in Acute Myocardial Infarction Patients Treated With Complete Revascularization Strategy |
| VANISH | Valsartan for Attenuating Disease Evolution in Early Sarcomeric Hypertrophic Cardiomyopathy |
| VESALIUS-CV | Effect of Evolocumab in Patients at High Cardiovascular Risk Without Prior Myocardial Infarction or Stroke |
| VICTOR | Vericiguat in Adults with Chronic Heart Failure and Reduced Ejection Fraction |
| VICTORIA | A Study of Vericiguat in Participants With Heart Failure With Reduced Ejection Fraction (HFrEF) (MK-1242-001) |
| ZENITH | A Study of Sotatercept in Participants With PAH WHO FC III or FC IV at High Risk of Mortality (MK-7962-006/​ZENITH) |

**References**

1. Kim D, Shim J, Choi E-K, Oh I-Y, Kim J, Lee YS, et al. Long-Term Anticoagulation Discontinuation After Catheter Ablation for Atrial Fibrillation: The ALONE-AF Randomized Clinical Trial. *JAMA* 2025;**334**:1246–1254.

2. Shoamanesh A, Field TS, Coutts SB, Sharma M, Gladstone D, Hart RG, et al. Apixaban versus aspirin for stroke prevention in people with subclinical atrial fibrillation and a history of stroke or transient ischaemic attack: subgroup analysis of the ARTESiA randomised controlled trial. *Lancet Neurol* 2025;**24**:140–151.

3. Piazza G, Bikdeli B, Pandey AK, Krishnathasan D, Khairani CD, Bejjani A, et al. Apixaban for Extended Treatment of Provoked Venous Thromboembolism. *N Engl J Med* 2025;**393**:1166–1176.

4. Shah JA, Hussain J, Ahmed B, Batra MK, Ali G, Naz M, et al. Rivaroxaban vs Warfarin in Acute Left Ventricular Thrombus Following Myocardial Infarction: RIVAWAR, An Open-Label RCT. *JACC Adv* 2025;**4**:101978.

5. Choi KH, Park YH, Lee J-Y, Jeong J-O, Kim CJ, Yun KH, et al. Efficacy and safety of clopidogrel versus aspirin monotherapy in patients at high risk of subsequent cardiovascular event after percutaneous coronary intervention (SMART-CHOICE 3): a randomised, open-label, multicentre trial. *Lancet* 2025;**405**:1252–1263.

6. Lee S-J, Yu HT, Lee Y-J, Lee S-H, Heo JH, Ahn SG, et al. Therapy for Atrial Fibrillation in Patients with Drug-Eluting Stents. *N Engl J Med* 2025.

7. Miao Z, Luo G, Song L, Sun D, Chen W, Yao X, et al. Intra-arterial Tenecteplase for Acute Stroke After Successful Endovascular Therapy: The ANGEL-TNK Randomized Clinical Trial. *JAMA* 2025;**334**:582–591.

8. Huang J, Yang J, Liu C, Li L, Yang D, Guo C, et al. Intra-Arterial Tenecteplase Following Endovascular Reperfusion for Large Vessel Occlusion Acute Ischemic Stroke: The POST-TNK Randomized Clinical Trial. *JAMA* 2025;**333**:579–588.

9. Munkhaugen J, Kristensen AMD, Halvorsen S, Holmager T, Olsen MH, Bakken A, et al. Beta-Blockers after Myocardial Infarction in Patients without Heart Failure. *N Engl J Med* 2025.

10. Procopi N, Zeitouni M, Kerneis M, Cayla G, Ferrari E, Range G, et al. Beta-blocker interruption effects on blood pressure and heart rate after myocardial infarction: the AβYSS trial. *Eur Heart J* 2025;**46**:2894–2902.

11. Lee CJ, Ihm S-H, Shin D-H, Jeong J-O, Kim JH, Chun K-H, et al. Spironolactone vs Amiloride for Resistant Hypertension: A Randomized Clinical Trial. *JAMA* 2025;**333**:2073–2082.

12. Wharton S, Lingvay I, Bogdanski P, Duque do Vale R, Jacob S, Karlsson T, et al. Oral Semaglutide at a Dose of 25 mg in Adults with Overweight or Obesity. *N Engl J Med* 2025;**393**:1077–1087.

13. Raposeiras-Roubin S, Amat-Santos IJ, Rossello X, González Ferreiro R, González Bermúdez I, Lopez Otero D, et al. Dapagliflozin in Patients Undergoing Transcatheter Aortic-Valve Implantation. *N Engl J Med* 2025;**392**:1396–1405.

14. Guo X, Sun G, Xu Y, Zhou S, Song Q, Li Y, et al. Benefit-harm trade-offs of intensive blood pressure control versus standard blood pressure control on cardiovascular and renal outcomes: an individual participant data analysis of randomised controlled trials. *Lancet* 2025;**406**:1009–1019.

15. Song Q, Peng X, Bai J, Yang R, Ling Q, Chen S, et al. Intensive Blood Pressure Control in Older Patients With Hypertension: 6-Year Results of the STEP Trial. *J Am Coll Cardiol* 2025:S0735-1097(25)07023-8.

16. Nicolau AM, Giugliano RP, Zimerman A, Afilalo J, Gencer B, Steffel J, et al. Outcomes in Older Patients After Switching to a Newer Anticoagulant or Remaining on Warfarin: The COMBINE-AF Substudy. *J Am Coll Cardiol* 2025;**86**:426–439.

17. Bea S, Iyer GS, Kim DH, Lin KJ, Zhang Y, Zakoul H, et al. Oral Anticoagulation and Risk of Adverse Clinical Outcomes in Venous Thromboembolism. *JAMA Intern Med* 2025;**185**:837–846.

18. Rossello X, Prescott EIB, Kristensen AMD, Latini R, Fuster V, Fagerland MW, et al. β blockers after myocardial infarction with mildly reduced ejection fraction: an individual patient data meta-analysis of randomised controlled trials. *Lancet* 2025;**406**:1128–1137.

19. Judge DP, Alexander KM, Cappelli F, Fontana M, Garcia-Pavia P, Gibbs SDJ, et al. Efficacy of Acoramidis on All-Cause Mortality and Cardiovascular Hospitalization in Transthyretin Amyloid Cardiomyopathy. *J Am Coll Cardiol* 2025;**85**:1003–1014.

20. Judge DP, Gillmore JD, Alexander KM, Ambardekar AV, Cappelli F, Fontana M, et al. Long-Term Efficacy and Safety of Acoramidis in ATTR-CM: Initial Report From the Open-Label Extension of the ATTRibute-CM Trial. *Circulation* 2025;**151**:601–611.

21. Masri A, Judge DP, Ruberg FL, Gillmore JD, Grodin JL, Obici L, et al. Effect of Acoramidis on Recurrent and Cumulative Cardiovascular Outcomes in ATTR-CM: Exploratory Analysis From ATTRibute-CM. *J Am Coll Cardiol* 2025:S0735-1097(25)07774-5.

22. Ostrominski JW, Claggett BL, Jerosch-Herold M, Raja AA, Day SM, Russell MW, et al. Valsartan and Cardiac Remodeling in Early-Stage Hypertrophic Cardiomyopathy: The VANISH Randomized Clinical Trial Cardiac Magnetic Resonance Substudy. *JAMA Cardiol* 2025;**10**:617–623.

23. Marx N, Deanfield JE, Mann JFE, Arechavaleta R, Bain SC, Bajaj HS, et al. Oral Semaglutide and Cardiovascular Outcomes in People With Type 2 Diabetes, According to SGLT2i Use: Prespecified Analyses of the SOUL Randomized Trial. *Circulation* 2025;**151**:1639–1650.

24. Packer M, Zile MR, Kramer CM, DiMaria JM, Baum SJ, Litwin SE, et al. Influence of Type 2 Diabetes on the Effects of Tirzepatide in Patients With Heart Failure and a Preserved Ejection Fraction With Obesity: A Prespecified Stratification-Based Analysis. *J Am Coll Cardiol* 2025;**86**:696–707.

25. Packer M, Zile MR, Kramer CM, Murakami M, Ou Y, Borlaug BA, et al. Interplay of Chronic Kidney Disease and the Effects of Tirzepatide in Patients With Heart Failure, Preserved Ejection Fraction, and Obesity: The SUMMIT Trial. *J Am Coll Cardiol* 2025;**85**:1721–1735.

26. Zile MR, Borlaug BA, Kramer CM, Baum SJ, Litwin SE, Menon V, et al. Effects of Tirzepatide on the Clinical Trajectory of Patients With Heart Failure, Preserved Ejection Fraction, and Obesity. *Circulation* 2025;**151**:656–668.

27. Borlaug BA, Zile MR, Kramer CM, Ye W, Ou Y, Hurt K, et al. Impact of Body Mass Index, Central Adiposity, and Weight Loss on the Benefits of Tirzepatide in HFpEF: The SUMMIT Trial. *J Am Coll Cardiol* 2025;**86**:242–255.

28. Jastreboff AM, Roux CW le, Stefanski A, Aronne LJ, Halpern B, Wharton S, et al. Tirzepatide for Obesity Treatment and Diabetes Prevention. *N Engl J Med* 2025;**392**:958–971.

29. Zimerman A, Kunzler ALF, Weber BN, Ran X, Murphy SA, Wang H, et al. Intensive Lowering of LDL Cholesterol Levels With Evolocumab in Autoimmune or Inflammatory Diseases: An Analysis of the FOURIER Trial. *Circulation* 2025;**151**:1467–1476.

30. Juhasz V, Drobni ZD, Quinaglia T, Gilman HK, Brendel JM, Suero-Abreu GA, et al. Atorvastatin and Aortic Stiffness During Anthracycline-Based Chemotherapy: A Secondary Analysis of a Randomized Clinical Trial. *JAMA Cardiol* 2025:e254548.

31. Pabon MA, Filippatos G, Claggett BL, Miao MZ, Desai AS, Jhund PS, et al. Finerenone Reduces New-Onset Atrial Fibrillation Across the Spectrum of Cardio-Kidney-Metabolic Syndrome: The FINE-HEART Pooled Analysis. *J Am Coll Cardiol* 2025;**85**:1649–1660.

32. Lemesle G, Didier R, Steg PG, Simon T, Montalescot G, Danchin N, et al. Aspirin in Patients with Chronic Coronary Syndrome Receiving Oral Anticoagulation. *N Engl J Med* 2025;**393**:1578–1588.

33. Munckhof A van de, Kammen MS van, Tatlisumak T, Krzywicka K, Aaron S, Antochi F, et al. Direct oral anticoagulants versus vitamin K antagonists for cerebral venous thrombosis (DOAC-CVT): an international, prospective, observational cohort study. *Lancet Neurol* 2025;**24**:199–207.

34. Khan J, Park KW, Han J-K, Hwang D, Yang H-M, Park S. Dual antiplatelet therapy after percutaneous coronary intervention according to bleeding risk (HOST-BR): an open-label, multicentre, randomised clinical trial. *The Lancet* 2026:p2244-2256.

35. Guimarães PO, Franken M, Tavares CAM, Antunes MO, Silveira FS, Andrade PB, et al. Early Withdrawal of Aspirin after PCI in Acute Coronary Syndromes. *N Engl J Med* 2025.

36. Verma A, Birnie DH, Jiang C, Heidbüchel H, Hindricks G, Kirchhof P, et al. Antithrombotic Therapy after Successful Catheter Ablation for Atrial Fibrillation. *N Engl J Med* 2025.

37. Warach SJ, Davis LA, Lawrence P, Gajewski B, Wick J, Shi F, et al. Optimal Delay Time to Initiate Anticoagulation After Ischemic Stroke in Atrial Fibrillation: A Pragmatic, Response-Adaptive Randomized Clinical Trial. *JAMA Neurol* 2025;**82**:470–476.

38. Jeppsson A, James S, Moller CH, Malm CJ, Dalén M, Vanky F, et al. Ticagrelor and Aspirin or Aspirin Alone after Coronary Surgery for Acute Coronary Syndrome. *N Engl J Med* 2025.

39. Hakeem A, Shah JA, Kumar R, Ali A, Lakho AA, Zeeshan H, et al. Twice-Daily Clopidogrel vs Ticagrelor to Reduce Short-Term Major Adverse Cardiovascular Events After Primary Percutaneous Coronary Intervention: The TADCLOT Trial. *J Am Coll Cardiol* 2025:S0735-1097(25)07549-7.

40. Kang D-Y, Wee S-B, Ahn J-M, Park H, Yun S-C, Park K-H, et al. Temporal modulation of antiplatelet therapy in high-risk patients undergoing complex percutaneous coronary intervention: the TAILORED-CHIP randomized clinical trial. *Eur Heart J* 2025:ehaf652.

41. Jolly SS, Entremont M-A d’, Lee SF, Mian R, Tyrwhitt J, Kedev S, et al. Colchicine in Acute Myocardial Infarction. *N Engl J Med* 2025;**392**:633–642.

42. Psaltis PJ, Nguyen MT, Singh K, Sinhal A, Wong DTL, Alcock R, et al. Optical coherence tomography assessment of the impact of colchicine on non-culprit coronary plaque composition after myocardial infarction. *Cardiovasc Res* 2025;**121**:468–478.

43. Jolly SS, Entremont M-A d’, Pitt B, Lee SF, Mian R, Tyrwhitt J, et al. Routine Spironolactone in Acute Myocardial Infarction. *N Engl J Med* 2025;**392**:643–652.

44. McDermott MM, Domanchuk KJ, Tian L, Zhao L, Zhang D, Bazzano L, et al. Metformin to Improve Walking Performance in Lower Extremity Peripheral Artery Disease: The PERMET Randomized Clinical Trial. *JAMA* 2025:e2521358.

45. Kristensen AMD, Rossello X, Atar D, Yndigegn T, Kimura T, Latini R, et al. Beta-Blockers after Myocardial Infarction with Normal Ejection Fraction. *N Engl J Med* 2025.

46. Anker SD, Friede T, Butler J, Talha KM, Placzek M, Diek M, et al. Intravenous Ferric Carboxymaltose in Heart Failure With Iron Deficiency: The FAIR-HF2 DZHK05 Randomized Clinical Trial. *JAMA* 2025;**333**:1965–1976.

47. Ibanez B, Latini R, Rossello X, Dominguez-Rodriguez A, Fernández-Vazquez F, Pelizzoni V, et al. Beta-Blockers after Myocardial Infarction without Reduced Ejection Fraction. *N Engl J Med* 2025.

48. Butler J, McMullan CJ, Anstrom KJ, Barash I, Bonaca MP, Borentain M, et al. Vericiguat in patients with chronic heart failure and reduced ejection fraction (VICTOR): a double-blind, placebo-controlled, randomised, phase 3 trial. *Lancet* 2025;**406**:1341–1350.

49. Fearon WF, Terada K, Takahashi K, Skoda A, Luikart HI, Lamendola CA, et al. Cardiac Allograft Vasculopathy Inhibition with Alirocumab: The CAVIAR Trial. *Circulation* 2025.

50. Verma S, Leiter LA, Teoh H, Mancini GBJ, Quan A, Elituv R, et al. Effect of evolocumab on saphenous vein graft patency after coronary artery bypass surgery (NEWTON-CABG CardioLink-5): an international, randomised, double-blind, placebo-controlled trial. *Lancet* 2025;**406**:1223–1234.

51. Desai MY, Owens AT, Abraham T, Olivotto I, Garcia-Pavia P, Lopes RD, et al. Mavacamten in Symptomatic Nonobstructive Hypertrophic Cardiomyopathy. *N Engl J Med* 2025;**393**:961–972.

52. Berg DD, Patel SM, Haller PM, Cange AL, Palazzolo MG, Bellavia A, et al. Dapagliflozin in Patients Hospitalized for Heart Failure: Primary Results of the DAPA ACT HF-TIMI 68 Randomized Clinical Trial and Meta-Analysis of Sodium-Glucose Cotransporter-2 Inhibitors in Patients Hospitalized for Heart Failure. *Circulation* 2025;**152**:1411–1422.

53. Rosenstock J, Hsia S, Nevarez Ruiz L, Eyde S, Cox D, Wu W-S, et al. Orforglipron, an Oral Small-Molecule GLP-1 Receptor Agonist, in Early Type 2 Diabetes. *N Engl J Med* 2025;**393**:1065–1076.

54. Wharton S, Aronne LJ, Stefanski A, Alfaris NF, Ciudin A, Yokote K, et al. Orforglipron, an Oral Small-Molecule GLP-1 Receptor Agonist for Obesity Treatment. *N Engl J Med* 2025;**393**:1796–1806.

55. Ji L, Jiang H, Bi Y, Li H, Tian J, Liu D, et al. Once-Weekly Mazdutide in Chinese Adults with Obesity or Overweight. *N Engl J Med* 2025;**392**:2215–2225.

56. Jastreboff AM, Ryan DH, Bays HE, Ebeling PR, Mackowski MG, Philipose N, et al. Once-Monthly Maridebart Cafraglutide for the Treatment of Obesity - A Phase 2 Trial. *N Engl J Med* 2025;**393**:843–857.

57. Garvey WT, Blüher M, Osorto Contreras CK, Davies MJ, Winning Lehmann E, Pietiläinen KH, et al. Coadministered Cagrilintide and Semaglutide in Adults with Overweight or Obesity. *N Engl J Med* 2025;**393**:635–647.

58. Davies MJ, Bajaj HS, Broholm C, Eliasen A, Garvey WT, Roux CW le, et al. Cagrilintide-Semaglutide in Adults with Overweight or Obesity and Type 2 Diabetes. *N Engl J Med* 2025;**393**:648–659.

59. Ruff CT, Patel SM, Giugliano RP, Morrow DA, Hug B, Kuder JF, et al. Abelacimab versus Rivaroxaban in Patients with Atrial Fibrillation. *N Engl J Med* 2025;**392**:361–371.

60. Piccini JP, Patel MR, Steffel J, Ferdinand K, Van Gelder IC, Russo AM, et al. Asundexian versus Apixaban in Patients with Atrial Fibrillation. *N Engl J Med* 2025;**392**:23–32.

61. Laffin LJ, Kopjar B, Melgaard C, Wolski K, Ibbitson J, Bhikam S, et al. Lorundrostat Efficacy and Safety in Patients with Uncontrolled Hypertension. *N Engl J Med* 2025;**392**:1813–1823.

62. Saxena M, Laffin L, Borghi C, Fernandez Fernandez B, Ghali JK, Kopjar B, et al. Lorundrostat in Participants With Uncontrolled Hypertension and Treatment-Resistant Hypertension: The Launch-HTN Randomized Clinical Trial. *JAMA* 2025;**334**:409–418.

63. Flack JM, Azizi M, Brown JM, Dwyer JP, Fronczek J, Jones ESW, et al. Efficacy and Safety of Baxdrostat in Uncontrolled and Resistant Hypertension. *N Engl J Med* 2025;**393**:1363–1374.

64. Desai AS, Karns AD, Badariene J, Aswad A, Neutel JM, Kazi F, et al. Add-On Treatment With Zilebesiran for Inadequately Controlled Hypertension: The KARDIA-2 Randomized Clinical Trial. *JAMA* 2025;**334**:46–55.

65. Van’t Hof AWJ, Gibson CM, Rikken S a. OF, Januzzi JL, Granger CB, Beurden A van, et al. Zalunfiban at First Medical Contact for ST-Elevation Myocardial Infarction. *NEJM Evid* 2025:EVIDoa2500268.

66. Hegde SM, Wang X, Garcia-Pavia P, Getchevski S, Masri A, Merkely B, et al. Effect of Aficamten Compared With Metoprolol on Echocardiographic Measures in Symptomatic Obstructive Hypertrophic Cardiomyopathy: MAPLE-HCM. *J Am Coll Cardiol* 2025:S0735-1097(25)07466-2.

67. Nissen SE, Ni W, Shen X, Wang Q, Navar AM, Nicholls SJ, et al. Lepodisiran - A Long-Duration Small Interfering RNA Targeting Lipoprotein(a). *N Engl J Med* 2025.

68. Nicholls SJ, Nelson AJ, Ditmarsch M, Kastelein JJP, Ballantyne CM, Ray KK, et al. Safety and Efficacy of Obicetrapib in Patients at High Cardiovascular Risk. *N Engl J Med* 2025;**393**:51–61.

69. Ballantyne CM, Gellis L, Tardif J-C, Banka P, Navar AM, Asprusten EA, et al. Efficacy and Safety of Oral PCSK9 Inhibitor Enlicitide in Adults With Heterozygous Familial Hypercholesterolemia: A Randomized Clinical Trial. *JAMA* 2025:e2520620.

70. Rosenson RS, López JAG, Gaudet D, Baum SJ, Stout E, Lepor NE, et al. Olpasiran, Oxidized Phospholipids, and Systemic Inflammatory Biomarkers: Results From the OCEAN(a)-DOSE Trial. *JAMA Cardiol* 2025;**10**:482–486.

71. Ray KK, Oru E, Rosenson RS, Jones J, Ma X, Walgren J, et al. Durability and efficacy of solbinsiran, a GalNAc-conjugated siRNA targeting ANGPTL3, in adults with mixed dyslipidaemia (PROLONG-ANG3): a double-blind, randomised, placebo-controlled, phase 2 trial. *Lancet* 2025;**405**:1594–1607.

72. Koren MJ, Vega RB, Agrawal N, Xu Y, Barbour AM, Yu H, et al. An Oral PCSK9 Inhibitor for Treatment of Hypercholesterolemia: The PURSUIT Randomized Trial. *J Am Coll Cardiol* 2025;**85**:1996–2007.

73. Li L, Zhou Y, Deng C, Sheng J, Peng D, Ling Z, et al. Recaticimab in adult heterozygous familial hypercholesterolaemia (REMAIN-3): a multicentre, randomized, double-blind, placebo-controlled Phase 3 study. *Cardiovasc Res* 2025;**121**:1856–1864.

74. Sarraju A, Brennan D, Hayden K, Stronczek A, Goldberg AC, Michos ED, et al. Fixed-dose combination of obicetrapib and ezetimibe for LDL cholesterol reduction (TANDEM): a phase 3, randomised, double-blind, placebo-controlled trial. *Lancet* 2025;**405**:1757–1768.
